# Genome-wide expert annotation of the epigenetic machinery of the plant-parasitic nematodes *Meloidogyne* spp., with a focus on the asexually reproducing species

**DOI:** 10.1186/s12864-018-4686-x

**Published:** 2018-05-03

**Authors:** Loris Pratx, Corinne Rancurel, Martine Da Rocha, Etienne G. J. Danchin, Philippe Castagnone-Sereno, Pierre Abad, Laetitia Perfus-Barbeoch

**Affiliations:** 1 Université Côte d’Azur, INRA, ISA, Sophia Antipolis, France; 2 Institut Sophia Agrobiotech, 400, route des chappes, BP 167 - 06903 Sophia Antipolis Cedex, France

**Keywords:** Adaptation, Epigenetics, Functional comparative annotation, Orthology, Chromatin, Histone modifications, Root-knot nematodes, Meloidogyne, Plant parasitism, Asexual reproduction

## Abstract

**Background:**

The renewed interest in epigenetics has led to the understanding that both the environment and individual lifestyle can directly interact with the epigenome to influence its dynamics. Epigenetic phenomena are mediated by DNA methylation, stable chromatin modifications and non-coding RNA-associated gene silencing involving specific proteins called epigenetic factors.

Multiple organisms, ranging from plants to yeast and mammals, have been used as model systems to study epigenetics. The interactions between parasites and their hosts are models of choice to study these mechanisms because the selective pressures are strong and the evolution is fast.

The asexually reproducing root-knot nematodes (RKN) offer different advantages to study the processes and mechanisms involved in epigenetic regulation. RKN genomes sequencing and annotation have identified numerous genes, however, which of those are involved in the adaption to an environment and potentially relevant to the evolution of plant-parasitism is yet to be discovered.

**Results:**

Here, we used a functional comparative annotation strategy combining orthology data, mining of curated genomics as well as protein domain databases and phylogenetic reconstructions.

Overall, we show that (i) neither RKN, nor the model nematode *Caenorhabditis elegans* possess any DNA methyltransferases (DNMT) (ii) RKN do not possess the complete machinery for DNA methylation on the 6th position of adenine (6mA) (iii) histone (de)acetylation and (de)methylation pathways are conserved between *C. elegans* and RKN, and the corresponding genes are amplified in asexually reproducing RKN (iv) some specific non-coding RNA families found in plant-parasitic nematodes are dissimilar from those in *C. elegans*. In the asexually reproducing RKN *Meloidogyne incognita*, expression data from various developmental stages supported the putative role of these proteins in epigenetic regulations.

**Conclusions:**

Our results refine previous predictions on the epigenetic machinery of model species and constitute the most comprehensive description of epigenetic factors relevant to the plant-parasitic lifestyle and/or asexual mode of reproduction of RKN. Providing an atlas of epigenetic factors in RKN is an informative resource that will enable researchers to explore their potential role in adaptation of these parasites to their environment.

**Electronic supplementary material:**

The online version of this article (10.1186/s12864-018-4686-x) contains supplementary material, which is available to authorized users.

## Background

Epigenetic modifications are heritable yet metastable and cannot be explained by changes in nucleotide sequence [1]. In eukaryotes, packaging of DNA into chromatin has profound effects on cellular processes that utilize DNA as template, including transcription, replication, recombination and repair [2]. The nucleosome is the fundamental unit of chromatin and it is composed of an octamer of histones around which 147 base pairs of DNA are wrapped [3]. Subsequent compaction leads to higher order structures including the formation of very dense arrays of nucleosomes observed in heterochromatin [4]. Despite being tightly packed, the chromatin appears to be highly plastic thanks to several factors that influence both its local and global architecture. Two mechanisms of epigenetic regulations are generally considered: methylation of DNA and post-translational modifications of histones [5, 6]. Besides these major processes, a growing body of evidence indicates that regulatory non-coding RNAs play an important role in epigenetic control [7]. Epigenetics research conducted so far has raised new evidence about how environmental factors can impact the mechanisms through which biological processes and functions are regulated (for review, [8, 9]). In that respect, a pivotal role of epigenetic mechanisms has been shown in controlling various strategies of pathogens to hijack host cell pathways [10, 11].

The root-knot nematode (RKN) *Meloidogyne incognita* is a plant parasite of major agricultural importance which reproduces exclusively by mitotic parthenogenesis [12]. Despite its clonal mode of reproduction, this nematode can adapt to unfavorable conditions. For instance, avirulent *M. incognita* strains controlled by a resistance gene are able to overcome plant resistance and become virulent on these plants [13]. Considering that the proportion of individuals with this phenotype rises gradually over generations and does not follow a Mendelian transmission [14, 15], it is possible that epigenetic changes could represent one of the main driving forces of evolution in this species [16].

Here, we study the epigenetic determinants possibly involved in the phenotypic plasticity of this parasite based on four sequenced RKN genomes [17–19], and information on core epigenetic proteins available in public databases [20]. So far, a structured source for such information is missing for RKN but also for the model nematode *C. elegans*.

We provide a manually curated annotation giving information about more than 3500 candidate epigenetic regulators in 20 species, including four RKN, five other nematodes and a set of model species of interest.

For *M. incognita* genes, we included expression data across several developmental stages. Such combination of functional annotation on RKN epigenetic factors is relevant to cover all possible mechanisms that could underlie the success of RKN pathogens.

## Methods

### Proteomes used for functional comparative annotation

For comparative annotation of epigenetic factors of the four RKN species (*M. incognita, M. javanica, M. arenaria, M. hapla*), we selected a total of 16 species from publicly available protein databases, including species with specific lifestyles (Table 1). Six model species, *C. elegans*, *Drosophila melanogaster*, *Homo sapiens*, *Saccharomyces cerevisae*, *Schizosaccharomyces pombe* and *Arabidopsis thaliana*, were selected because of their genome completeness, annotation quality and the existence of experimental characterization of epigenetic factors. We included 10 other species based on their phylogenetic position and/or lifestyles: two other plant-parasitic nematodes (*Globodera pallida, G. rostochiensis*), two animal-parasitic nematodes (*Trichinella spiralis, Ascaris suum*)*,* and two plant-parasitic fungi with previous report of epigenetic regulations (*Botrytis cinerea, Leptosphaeria maculans* [21, 22])*.* Finally, we added four species which possess an epigenome of interest, known to be involved in phenotypical plasticity (*Acyrthosiphon pisum, Apis mellifera* [23, 24]) or in pathogenicity (*Schistosoma mansoni, Plasmodium falciparum* [25–28]). The 20 proteomes were all downloaded from Uniprot database [29], with the exception of *Meloidogyne* spp. available at "Meloidogyne genomic resources" website(https://meloidogyne.inra.fr) [19] and *Globodera* spp. downloaded from WormBase ParaSite [30, 31].

**Table 1:**
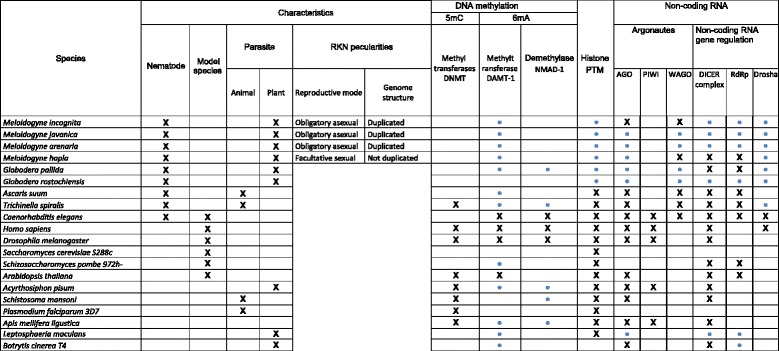
Characteristics of the 20 species used for functional comparative annotation of core epigenetic factors

A cross indicates either if the characteristic is true or if data about epigenetic mechanism is available for one species. Blue circles indicate new core epigenetic factors annotated in this study

### Identification of core epigenetic factors

**Fig. 1:**
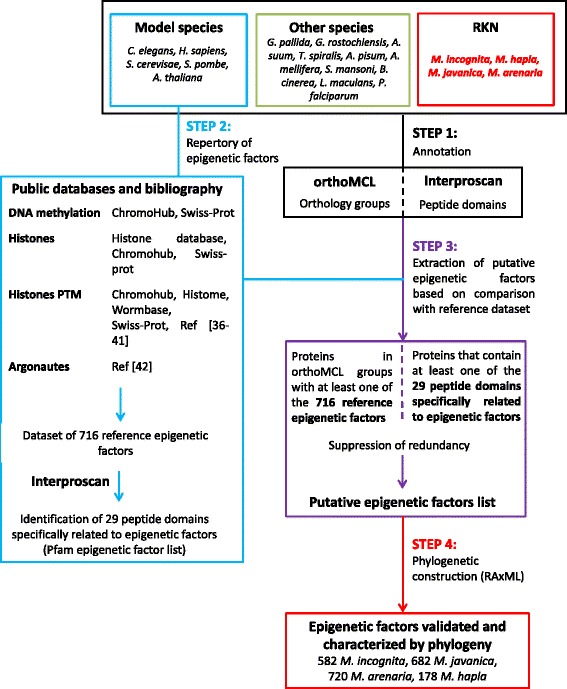
Pipeline for annotation of core epigenetic factors with a focus on root-knot nematodes (RKN). Step 1 consisted of the annotation, based on both OrthoMCL and Interproscan, of 4 RKN species together with 6 model species and 10 other species selected for their lifestyles and/or epigenetic interest. In parallel, step 2 consisted of creating a repertory of known epigenetic factors from public data on 6 model species. Step 3 consisted of the extraction of putative epigenetic factor from step 1 data, based on comparison with reference dataset created during step 2. Finally, step 4 consisted of the validation and characterization of epigenetic factors thanks to phylogenetic analysis

Epigenetic factors were identified thanks to a custom pipeline including four annotation steps (Fig. 1). The first step consisted in the identification of both (i) orthology links between the 20 proteomes by using OrthoMCL version 2.0 under standard parameters [32], and (ii) search of specific Pfam [33] protein domains assigned by Interproscan [34]. To explore and analyze homology clusters we used a web server called Family-Companion [35].

As a second step, we created a repertory of known epigenetic factors and associated protein domains from the 6 model species. Basically, we recovered all known chromatin factors based on (i) Swiss-Prot [29] and Wormbase [36] annotations, (ii) epigenetic factors-specific databases such as Histone database [37], ChromoHub [38] and Histome [39], and (iii) literature [40–46]. This dataset of epigenetic factors constituted the reference dataset that we used for comparative genome annotation of RKN (Additional file 1: Table S1). Then, from this reference dataset, we looked for protein domains specifically associated with known epigenetic factors that could be used for functional annotation. These features were provided by Pfam database of curated protein families [47–49]. We only kept Pfam domains that could uniquely be linked to epigenetic factors (thereafter called “Pfam epigenetic factor list”; Additional file 2: Table S2). For instance, PCAF (P300/CBP-associated factor) N-terminal domain (PF06466) and acetyltransferase 1 domain (PF00583) were both associated with Gcn5-related N-acetyltransferases (GNAT) family of histone acetyltransferase. However, while PCAF N-terminal domain (PF06466) was restricted to histone acetyltransferases, we found acetyltransferase 1 domain (PF00583) also carried by proteins that do not have an activity on histone (for instance choline acetyltransferases). In that case, only PCAF N-terminal domain (PF06466) was kept for functional annotation.

The goal of the third step was to identify epigenetic factors among OrthoMCL groups and/or Interproscan annotation, from step one. Proteins orthologous to at least one protein of the reference dataset and/or containing at least one Pfam domain of interest were annotated as putative epigenetic factors. Any protein not positioned into any OrthoMCL group was called “singleton” as it possessed no evident ortholog or in-paralog.

To validate the accuracy of the annotation, and to determine the closest orthologs between *C. elegans* and RKN, we performed phylogenetic constructions as the fourth step. Putative epigenetic factors protein sequences were aligned by MAFFT version 7.245 [50] with --auto option. The alignment was manually checked to remove misaligned sequences. We used trimAl version 1.2 [51] to remove gap-rich columns in the multiple alignments. Maximum likelihood trees were built with RAxML version 8.1 [52] with an automatic detection of the fittest evolutionary model, and an estimated gamma distribution of the rates of evolution (PROTGAMMAUTO option). Rapid bootstrap replicates followed by a full ML analysis were conducted. We used the -autoMRE criterion to stop bootstrap replicated upon convergence. For each phylogeny, the best scoring ML tree with associated bootstrap support values was retrieved as final resulting ML topology. The trees were visualized with FigTree version 1.4.2 [53].

### Expression analysis

To enhance robustness of the expert annotation, we integrated an experimental transcript verification step. We focused on *M. incognita* because RNA-seq data were available on six developmental stages of this species in our lab [54]. For each *M. incognita* putative epigenetic factor, reads per kilobase of transcript per million mapped reads (RPKM) was calculated. We considered that putative epigenetic factor annotations were supported by existence of transcripts when RPKM≥; 5.

## Results

Epigenetic factors were identified thank to a comparative annotation based on 6 model species. We selected 716 proteins, to build the reference dataset of epigenetic factors. Among these 716 proteins, 288 were from *H. sapiens*, 116 from *C. elegans*, 64 from *D. melanogaster*, 49 form *S. cerevisae*, 44 from *S. pombe* and 155 from *A. thaliana* (Additional file 1: Table S1 and Additional file 2: Table S2). Within this reference dataset, 29 Pfam domains were found to be specific to epigenetic factors (i.e. not found outside epigenetic factors) (Additional file 2: Table S2).

### The methodology is effective for the identification of epigenetic factors

To assess the efficiency of this annotation methodology, we tested the pipeline (Fig. 1) on two species, *S. mansoni* and *A. pisum*, for which epigenetic factors have already been annotated [23–27].

In *S. mansoni*, 56 of the 67 previously identified epigenetic factors (84%) could be identified with this methodology (Additional file 3: Table S3). Among the 11 missing *S. mansoni* proteins, one of them was positioned in the same OrthoMCL group as the human protein SNW1, which is not described to be a chromatin factor (Uniprot accession number: Q13573). Moreover, we were able to identify 36 proteins as new putative chromatin epigenetic factors in *S. mansoni*. In *A. pisum*, 82 of the 99 previously described epigenetic factors (83%) could be identified with our methodology (Additional file 4: Table S4). Four of the 17 missing *A. pisum* proteins were found as orthologs of non-chromatin factors. Moreover, we identified 150 proteins as new putative epigenetic factors of *A. pisum*. Taken together, these results show that this methodology allowed identification of the majority of known epigenetic factors in two non-model species and identified new putative epigenetic factors.

**Fig. 2:**
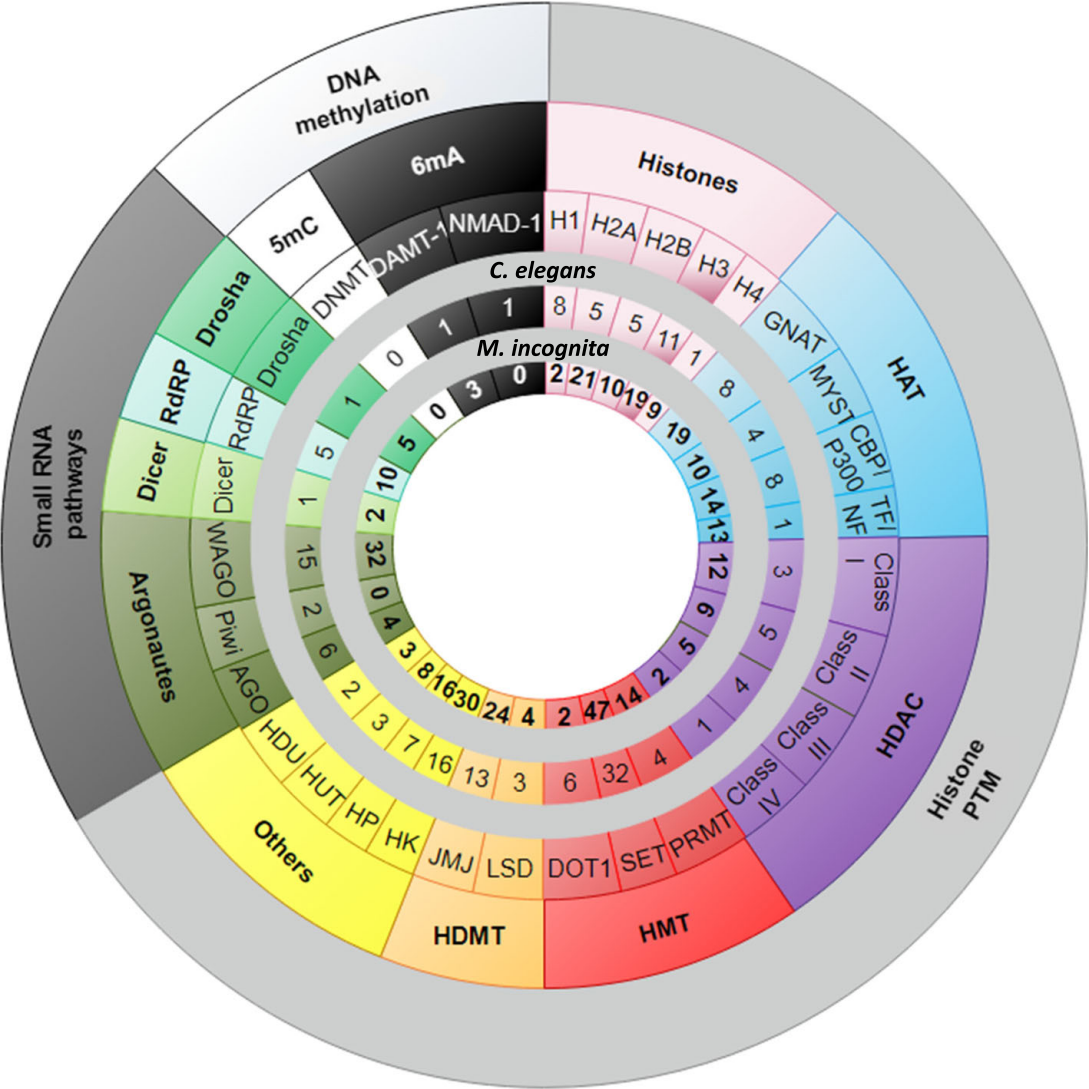
Number of epigenetic factors, classified by process, identified in *M. incognita* compared to *C. elegans*

When applied to RKN, this annotation methodology identified putative core epigenetic factors orthologous to those of the model nematode *C. elegans* and 15 other species. For each family, numbers of core epigenetic factors were identified in *M. incognita*, and then were compared to those of *C. elegans* (Fig. 2; Additional file 5: Table S5). Annotations for each of the three epigenetic processes are detailed below.

### Cytosine methylation machinery is absent in RKN and in all tested nematodes with the exception of *T. spiralis*

In both plants and animals, methylation on the fifth carbon of cytosine (5mC) is common and has been extensively studied [55]. DNA methyltransferases (DNMT) were searched by orthology links with the 14 reference DNMT (Additional file 1: Table S1 and Additional file 2: Table S2) and on the basis of DNA methyltransferase Pfam domain presence (PF00145). No DNMT could be identified in any plant-parasitic nematode (PPN). DNMT2 orthologs were identified in *A. suum* and *T. spiralis*, and DNMT3 was restricted to *T. spiralis*. Another putative DNMT (Uniprot reference: H2L057) was identified in *C. elegans* but could not be assigned to any DNMT family. H2L057 was identified by the presence of a DNA methyltransferase Pfam domain (PF00145) and was not associated to any orthoMCL group (“singleton”).

No DNMT1 was identified in any of the nine nematodes studied (*C. elegans, M. incognita, M. javanica, M. arenaria, M. hapla, G. pallida, G. rostochiensis, T. spiralis and A. suum*). However, according to the literature, DNMT1-like proteins were previously identified in nematodes, including *C. elegans*, *T. spiralis*, *A. suum* and *M. hapla* [16, 56]. We looked for these proteins in our dataset. *A. suum* DNMT1-like could not be found in Uniprot with the accession number given by the authors (GS_06989). *C. elegans* (Q9U1S4) and *T. spiralis* (E5SAG0) DNMT1-like proteins were not assigned to any OrthoMCL group (“singleton”) and did not possess the specific Pfam domain for DNMT (PF00145). *M. hapla* (Mhap1s0004g00613) DNMT1-like was positioned in an OrthoMCL group (GRP7217) containing 12 proteins from PPN and none of these proteins possessed the specific Pfam domain for DNMT (PF00145).

**Fig. 3:**
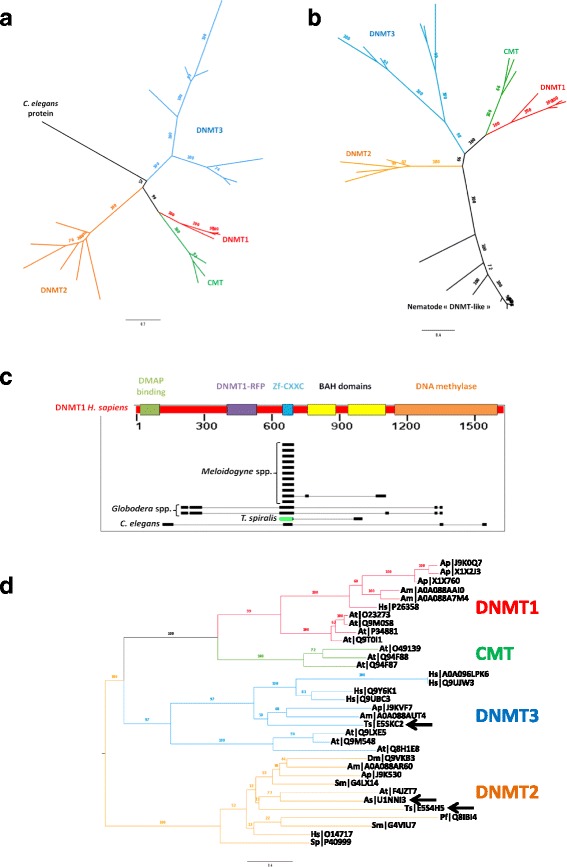
DNA-methyltransferases (DNMT) and chromomethylases (CMT) annotation. Phylogenetic tree was built with **a** putative DNMT and CMT sequences from 6 model species and 9 nematodes without a priori, or **b** putative DNMT and CMT sequences from 6 model species and nematode DNMT1-like orthologs previously identified by Gao et al., 2012. **c** Alignment of nematode DNMT1-like against *H. sapiens* DNMT1. **d** Corrected phylogenetic tree for DNMT and CMT sequences from the 20 species. Nematode DNMT are indicated by arrows

We built a phylogeny containing all DNMT, including the putative *C. elegans* DNMT (H2L057) and other nematodes DNMT1-like. Both the putative *C. elegans* DNMT and other nematodes DNMT-1 like grouped outside the known DNMT (Fig. 3a and b). Furthermore, the analysis of the protein domains carried by the DNMT1-like proteins showed they lack a DNA methylase domain (PF00145) and only contain a Pfam Zf-CXXC domain (PF02008) (Fig. 3c). Zf-CXXC domain is known to be associated to the recognition and binding to unmethylated cytosines in CpG. *H. sapiens* DNMT1 carries six protein domains, including a Zf-CXXC domain in position 646–691 and a DNA methylase domain in position 1140–1593. All nematode DNMT1-like could be aligned on a window from 635 to 693 in *H. sapiens* DNMT1, exactly on the Zf-CXXC domain position. However, the alignment of *Globodera spp*. and *C. elegans* DNMT1-like on the DNA methylase domain of *H. sapiens* DNMT1 never exceed 18 aminoacids while this domain size is 453 aminoacids, indicating that PPN, *Meloidogyne* spp. and *Globodera* spp., as well as *C. elegans*, do not possess any possibly functional DNMT. To date, in nematodes, only DNMT2 (*A. suum* and *T. spiralis*) and DNMT3 (*T. spiralis*) orthologs were found. Here, we provide an updated DNMT phylogeny in Fig. 3d.

### Adenine methylation (6mA) machinery is incomplete in RKN

Methylation of DNA on the sixth position of adenine (m6A) is involved in epigenetic transgenerational inheritance [57–59]. DNA N6-methyltransferases (DAMT) were searched by orthology links with the *C. elegans* reference DAMT-1 (Q09956; Table 2) and on the basis of S-adenosylmethionine-binding Pfam domain presence (PF05063). One ortholog of *C. elegans* DAMT-1 gene is found in *M. hapla* whereas multiple co-orthologs are found in the three asexually reproducing RKN species, *M. incognita, M. javanica, M. arenaria* (Table 2 and Additional file 6: Figure S1). One *M. incognita* ortholog, among the three putative Mi-DAMT-1 identified, was supported by transcriptomic data (Fig. 5). Then, we looked for 6mA demethylases by orthology links with the 5 *C. elegans* AlkB family members (Alkylated DNA repair protein B), together with the presence of AlkB family Pfam domain (PF13532). Proteins of the AlkB family catalyze the demethylation of methylated nucleotides from both DNA and RNA. However, until now, only NMAD-1 (Q8MNT9; Table 2) has been shown with 6mA demethylase activity in *C. elegans* [58]. No ortholog of *C. elegans* NMAD-1 was found in the Meloidogyne spp. (Table 2 and Additional file 7: Figure S2).

**Table 2:** Summary of 6mA DNA methyltransferases and demethylases

Type	Family	Lineage			RKN				
			Ce		Mi		Mj	Ma	Mh
			Number	Sequences	Number	Transcriptional Support	Number	Number	Number
Methyltransferase	MTA70 like	DAMT-1	1	DAMT-1 /Q09956	3	X	3	4	1
Demethylases	AlkB	NMAD-1	1	NMAD1 /Q8MNT9	0		0	0	0
		AlkBH	3	alkb-8 /Q9U3P9 E0DBL0 Q7YWP5	7	X	11	10	2

In the model species *C. elegans* (Ce), the number, the names and the Uniprot accession numbers of genes are indicated. Number of orthologs in RKN (*M. incognita*, Mi; *M. javanica*, Mj; *M. arenaria*, Ma; *M. hapla*, Mh) is indicated for each lineage. For *M. incognita*, presence of RNA-seq transcriptional support is indicated by a cross. DAMT, DNA N6-methyl methyltransferase; NMAD, DNA N6-methyl adenine demethylase; AlkB, Alkylated DNA repair protein B; AlkBH, Alkylated DNA repair protein B homolog

### Histones and histone modifying-enzymes are conserved in RKN

**Fig. 4:**
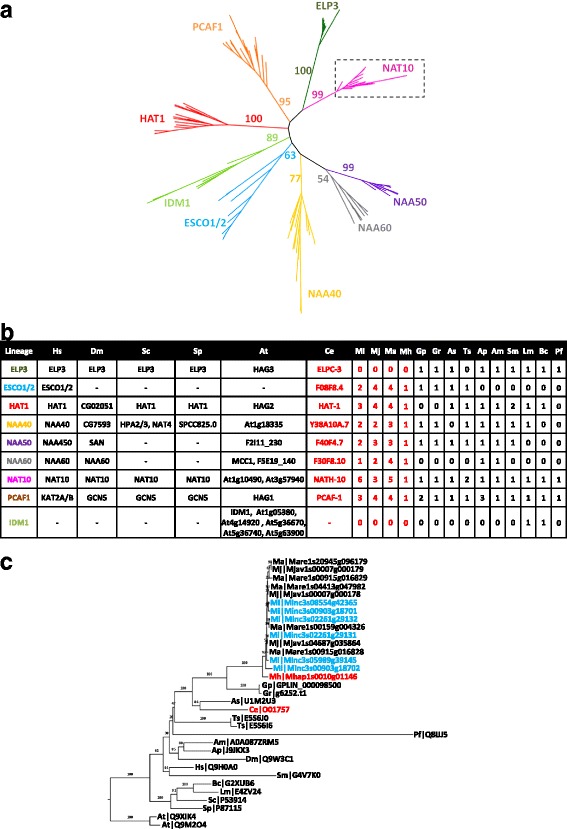
Representative example of histone modifying enzymes phylogenetic tree: GNAT histone acetyltransferase tree. GNAT histone acetyltransferases were identified in 20 species of interest. **a** Phylogenetic tree of all the 20 species GNAT histone acetyltransferases shows that each epigenetic factor protein fit into specific lineages (ELP3, ESCO1/2, HAT1, NAA40, NAA50, NAA60, NAT10, PCAF1 and IDM1). **b** Number of epigenetic factor protein for each lineage identified in the 20 species. **c** Close up of the NAT10 lineage

Histones and histone modifying-enzymes were sought by either orthology links with the 631 reference proteins and/or on the basis of at least one of the 21 associated-Pfam domain presence (Additional file 1: Table S1 and Additional file 2: Table S2). This strategy led to the identification of 573 histones and candidate histone modifying-enzymes in *M. incognita*, 730 in *M. javanica*, 771 in *M. arenaria* and 177 in *M. hapla*. All known histones (H1, H2A, H2B, H3 and H4) and families of histone modifying-enzymes (Histone Acetyltransferases, HAT; Histone deacetylases, HDAC; Histone methyltransferases, HMT; Histone demethylases, HDMT; Histone kinases, HK; Histone phosphatases, HP; Histone ubiquitinyl-transferases, HUT; Histone deubiquitinases, HDU) could be identified (Table 3; Additional file 8: Table S6). Then, we focused on histone acetylation and histone methylation and built phylogenies to associate every *Meloidogyne* spp. putative epigenetic factor to its closest ortholog in *C. elegans* (Fig. 4 and Additional file 9: Figure S3, Additional file 10: Figure S4, Additional file 11: Figure S5, Additional file 12: Figure S6, Additional file 13: Figure S7, Additional file 14: Figure S8, Additional file 15: Figure S9 and Additional file 16: Figure S10). For instance, the phylogenetic tree of GNAT family is shown in Fig. 4. This tree is representative of what was found in all families. Members of the GNAT family were found in all 20 species (Fig. 4a and b). A close up on the NAT10 lineage showed that when one gene is present in *C. elegans*, one ortholog is found in *M. hapla* whereas multiple co-orthologs are found in the three asexually reproducing RKN species, *M. incognita, M. javanica, M. arenaria* (Fig. 4b and c). In summary, this approach led to the identification of 65 protein lineages that contained at least one *C. elegans* protein. Among these 65 protein lineages, 48 (74%) were associated with at least one epigenetic factor of RKN (Table 3; Additional file 8: Table S6).

**Table 3:** Summary of histones and histone modifying enzymes annotation

Type	Family	Lineage	Model species				RKN				
			Ce		Hs		Mi		Mj	Ma	Mh
			Number	Sequences	Number	Sequences	Number	Transcriptional Support	Number	Number	Number
Histone	Linker	H1	9	HIL-1/2/3/4/5/6/7/8/, HIS-24	11	H1, H1.0/1/2/3/4/5, H1F0, H1t, H1oo, H1x	2	X	7	10	2
	Core	H2A	5	HIS-3/35, HTAS-1, HTZ-1, ZC155.2	18	H2A.1, H2A.J, H2A.V, H2A.Z, H2A1A/B/D/H/J, H2A2A/B/C, H2A3, H2AB1, H2AFB1, H2AG, H2AX, H2AW	21	X	28	23	5
		H2B	5	HIS-4/11/39/41/48	22	H2B1A/B/C/D/E/F/G/H/I/K/L/M/N/O, H2B2C/D/E/F, H2B3B, H2BFM, H2BFS, H2BWT	10	X	12	16	1
		H3	11	CPAR-1, HCP-3, HIS-2/40/69/70/71/72/73/74, F20D6.9	6	H3, H3.1/2/3, H3.1 t, H3.3C	19	X	22	23	7
		H4	1	HIS-1	2	H4, H4G	9	X	11	9	0
Histone acetyltransferase	GNAT	ELP3	1	ELPC-3	1	ELP3	0		0	0	0
		F08F8.4	1	F08F8.4	2	ESCO1/2	2	X	4	4	1
		HAT1	1	HAT-1	1	HAT1	3	X	4	4	1
		NAA40	1	Y38A10A.7	1	NAA40	2	X	2	3	1
		NAA50	1	F40F4.7	1	NAA50	2	X	3	3	1
		NAA60	1	F30F8.10	1	NAA60	1		2	4	1
		NAT10	1	NATH-10	1	NAT10	6	X	3	5	1
		PCAF1	1	PCAF-1	1	KAT2A/B	3		4	4	1
	MYST	MYS1	1	MYS-1	1	KAT5	2	X	2	5	1
		MYS2	1	MYS-2	1	KAT8	3	X	5	4	1
		MYS4/LSY12 (LSY12)	1	LSY-12	2	KAT6A/B	3	X	6	3	1
		MYS4/LSY12 (MYS4)	1	MYS-4	0	–	2	X	3	8	1
	TF/NF	TAF1	1	TAF-1	2	TAF1/1 L	13	X	14	12	1
Histone deacetylase	Class I	Class1 (HDA1/3)	2	HDA-1/3	2	HDAC1/2	8	X	8	9	2
		Class1 (HDA2)	1	HDA-2	1	HDAC3	4	X	3	3	1
	Class II	Class II (HDA4)	1	HDA-4	4	HDAC4/5/7/9	4	X	3	4	1
		Class II (HDA5/6/10)	1	HDA-6	0	–	0		0	0	0
		Class II (HDA5/6/10)	3	HDA-5/10, F43G6.17	2	HDAC6/10	3	X	5	2	1
	Class III	Class III (SIR2.1)	1	SIR-2.1	1	SIRT1	3		3	3	1
		Class III (SIR2.2/2.3)	2	SIR-2.2/2.3	1	SIRT4	0		0	0	0
		Class III (SIR2.4)	1	SIR-2.4	1	SIRT6	2	X	1	2	1
	Class IV	HDA11	1	HDA-11	1	HDAC11	2	X	5	4	1
Histone methyltransferase	DOT1	DOT1	6	DOT-1.1-1.5, D1053.2	1	DOT1M	2	X	9	7	1
	PRMT	CARM1	0	–	1	CARM1	3	X	4	5	1
		PRMT1	1	PRMT-1	2	PRMT1/8	2	X	3	5	1
		PRMT3/7	1	PRMT-3	1	PRMT7	3	X	5	4	1
		PRMT3/7	1	PRMT-7	1	PRMT9	0		0	0	0
		PRMT5	1	PRMT-5	1	PRMT5	6		4	5	2
	SET eu1	SET30	1	SET-30	2	SMYD2/3	2	X	5	4	1
		SET3	1	SET-3	1	SETD7	0		0	0	0
		SET27	1	SET-27	0	–	0		0	0	0
		SET29	1	SET-29	0	–	3		4	4	1
	SET eu2	SET1	1	SET-1	0	–	5		7	7	2
		MES4	1	MES-4	2	NSD1, WHSC1L1	4	X	5	4	1
		SET16	1	SET-16	2	KMT2C/D	10	X	10	9	1
		SET2	1	SET-2	2	SETD1A/B	4	X	7	7	1
		Y73B3A.1	1	Y73B3A.1	1	KMT2E	2	X	3	3	0
		–	1	SET-8	0	–	0		0	0	0
		MET-1/LIN-59	1	MET-1	1	SETD2	0		0	0	0
		MET-1/LIN-59	1	LIN-59	1	ASH1L	0		0	0	0
	SET hetero	DNMT1/CMT (DNMT1)	0	–	2	SUV39H1/2	2		2	5	1
		MET2	1	MET-2	2	SETDB1/2	3	X	7	7	1
		SET23	1	SET-23	0	–	2	X	4	4	1
		MES2/SET12	1	MES-2	2	EZH1/2	3	X	5	5	1
		MES2/SET12	1	SET-12	1	WHSC1	0		0	0	0
		SET4	1	SET-4	2	KMT5B/C, Dm|Hmt4–2	2	X	5	6	1
		SET-11	1	SET-11	2	EHMT1/2	0		0	0	0
		SET-6 family	8	SET-6/13/15/19/20/21/32/33	0	–	0		0	0	0
		no	1	SET-25	0	–	0		0	0	0
		no	1	SET-9	0	–	0		0	0	0
	SET - PPN	Cons07	0	–	0	–	3	X	3	4	1
		Cons12	0	–	0	–	2	X	3	4	1
		Cons18	0	–	0	–	0		1	0	0
Histone deacetylase	LSD	AMX1	1	AMX-1	1	KDM1B	0		0	0	0
		LSD1/SPR5	2	LSD-1, SPR-5	1	KDM1A	4	X	6	6	2
	JMJ	JMJC1	1	JMJC-1	1	NO66	6	X	7	8	2
		JMJD1/JHDM1 (JHDM1)	1	JHDM-1	2	KDM2A/B	3	X	3	5	0
		JMJD1/JHDM1 (JMJD1)	2	JMJD-1.1/1.2	3	KDMT7A, PHF2, PHF8	2	X	3	5	0
		JMJD2	1	JMJD-2	5	KDM4A/B/C/D/E	3	X	9	10	1
		RBR2	1	RBR-2	4	KDM5A/B/C/D	4		6	5	1
		JMJD3/UTX1 (JMJD3)	3	JMJD-3.1/3.2/3.3	0	–	0		0	0	0
		JMJD3/UTX1 (UTX1)	1	UTX-1	3	KDM6A/B, UTY	2	X	2	4	1
		JMJD4/PSR1 (JMJD4)	1	JMJD-4	0	–	0		0	0	0
		JMJD5	1	JMJD-5	1	KDM8	2	X	2	2	1
		JMJD4/PSR1 (PSR1)	1	PSR-1	1	JMJD6	2	X	2	3	1

In the 2 model species (*C. elegans,* Ce; *H. sapiens,* Hs), the number and the names of genes are indicated. Number of orthologs in RKN (*M. incognita,* Mi; *M. javanica, *Mj; *M. arenaria,* Ma; *M. hapla,* Mh) is indicated for each lineage. For *M. incognita*, presence of RNA-seq transcriptional support is indicated by a cross. Substrates for the histone modifying enzymes of model species are indicated. Only the protein lineages tested by phylogeny are shown

**Fig. 5:**
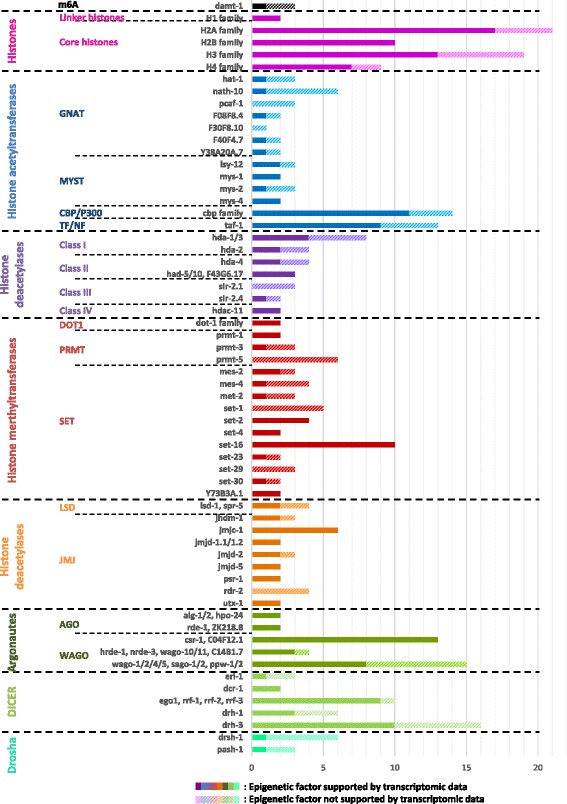
*M. incognita* putative epigenetic factors supported by RNA-seq. Number of *M. incognita* putative epigenetic factors are classified according to *C. elegans* orthology. For each *C. elegans* epigenetic factor, the number of *M. incognita* orthologs is indicated. Dark histograms indicated that the *M. incognita* gene is expressed (RPKM≥ 5) whereas light histograms indicated that there is no RNA-seq support of gene expression (RPKM< 5)

To assess the robustness of this annotation, we looked for transcriptional support of these putative histones and histone modifying enzymes based on *M. incognita* RNA-seq data. We found that most (42/65; 65%) of *C. elegans* lineages possessed at least one ortholog in *M. incognita* supported by transcriptomic data (Fig. 5). These results suggest that most of *C. elegans* histone modifying-pathways are conserved and expressed in *M. incognita*.

Furthermore, histone modifying enzymes without orthology to *C. elegans* were identified in RKN. Some of them were orthologs of other model species enzymes (Table 3; Additional file 8: Table S6). For instance, three *M. incognita* proteins were co-orthologs of human CARM1 (Histone-arginine methyltransferase 1). Candidate histone methyltransferases restricted to PPN (*Globodera* spp. and *Meloidogyne* spp.) were also identified based on the presence of the characteristic SET Pfam domain (PF00856) and the lack of BLASTp hit against the NCBI nr database. They were called SET-PPN because they belong to the SET family of HMT and are, so far, specific to PPN. In *M. incognita*, these SET-PPN were supported by transcriptomic data (Table 3; Additional file 8: Table S6), suggesting they are functional.

### Small non-coding RNA epigenetic machinery exist in RKN but some components are missing

**Fig. 6:**
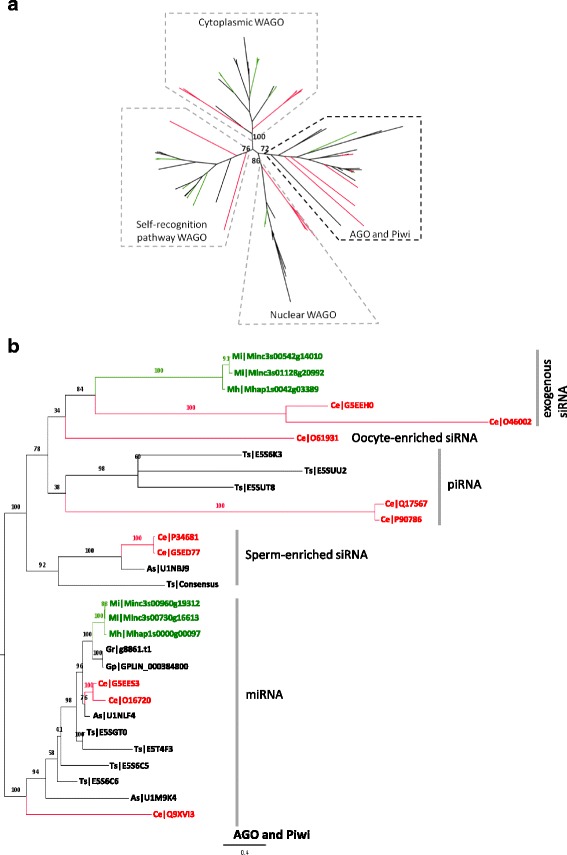
Argonaute phylogenetic tree. Putative Argonaute proteins from 7 nematodes (*C. elegans, M. incognita, M. hapla, G. pallida, G. rostochiensis, A. suum* and *T. spiralis*) were selected to build (**a**) whole Argonaute phylogenetic tree. From this whole Argonaute phylogenetic tree, four branches could be distinguished. One correspond to AGO and Piwi family of Argonautes (**b**) and three to WAGO (cytoplasmic WAGO, nuclear WAGO, self-recongnition pathway WAGO) Argonautes (Additional file 17: Figure S11). *M. incognita* and *M. hapla* are colored in green. *C. elegans* proteins are colored in red. Other nematodes are colored in black

Small non coding RNA (ncRNA) play a fundamental role in functional plasticity in eukaryotes [60]. In a general way, small ncRNAs serve as guide to Argonaute proteins (AGO) to regulate their respective targets for gene silencing. AGO proteins are characterized by the presence of PAZ (PF02170) and PIWI (PF02170) domains and can be classified into three clades ([46]; Table 3): (i) the AGO clade, which includes *A. thaliana* AGOs, human AGOs 1–4 and the *C. elegans* miRNA effectors ALG1/2; (ii) the PIWI clade, which includes the *C. elegans* PRG-1 and ERGO-1 (iii) an expanded family of worm-specific AGOs (WAGOs). To identify AGO proteins in RKN, we combined OrthoMCL groupings to the presence of Piwi domain (PF02171), PAZ domain (PF02170) and AGO1 domain (PF08699) [60]. To simplify the construction of the phylogeny, *M. incognita* and *M. hapla* were the only RKN tested because of the high similarity between *M. incognita* and the two other obligatory asexually reproducing RKN (*M. javanica*, *M. arenaria*). A total of 36 putative Argonautes was identified in *M. incognita* and 14 in *M. hapla* (Table 4). All of them could be associated to a specific non-coding RNA pathway, microRNA (miRNA) or small interfering RNA (siRNA), based on phylogeny (Fig. 6a). We could identify orthologs of *C. elegans* Argonautes involved in miRNA (ALG-1/2, HPO-24) and exogenous siRNA (RDE-1, ZK218.8) pathways (Fig. 6b). We could also identify all families of WAGO Argonautes (cytoplasmic WAGOs, nuclear WAGOs and the WAGO involved in self-recognition CSR-1; Additional file 17: Figure S11). Furthermore, WAGOs involved in self-recognition pathway seems amplified in RKN as we could identify 13 CSR-1-like in *M. incognita* and six in *M. hapla* (Table 4 and Additional file 17: Figure S11). All of the identified *M. incognita* Argonautes are expressed except for cytoplasmic WAGO for which only half of the genes have transcriptional supports (Fig. 5). In contrast, we could not identify any ortholog of *C. elegans* Argonautes involved in endogenous siRNA (Argonautes triggering sperm-enriched siRNA, ALG-3/4) nor Argonautes involved in piwiRNA (piRNA) pathways (Table 4, Fig. 6b and Additional file 18: Figure S12).

**Table 4:** Argonautes annotation

Type	Family	Lineage			RKN			Other nematodes			
			*C. elegans*		Mi		Mh	Gp	Gr	As	Ts
			Number	Sequences	Number	Transcriptional support	Number	Number	Number	Number	Number
Argonaute	AGO	miRNA	3	ALG-1, ALG-2, HPO-24	2	X	1	1	1	2	4
		sperm-enriched siRNA	2	ALG-3, ALG-4	0		0	0	0	1	86
		exogenous siRNA	2	RDE-1, ZK218.8	2	X	1	0	0	0	0
	PIWI	oogenesis-enriched siRNA	1	ERGO-1	0		0	0	0	0	0
		piRNA	1	PRG-1, (PRG-2)	0		0	0	0	0	3
	WAGO	Cytoplasmic WAGO	8	WAGO-1, WAGO-2, WAGO-4, WAGO-5, SAGO-1, SAGO-2, PPW-1, PPW-2	15	X	4	6	9	1	0
		Nuclear WAGO	5	HRDE-1, NRDE-3, WAGO-10, WAGO-11, C14B1.7	4	X	2	5	5	0	0
		Self recognition pathway	2	CSR-1, C04F12.1	13	X	6	7	9	1	0

In *C. elegans*, the number and the names of Argonautes are indicated. Number of orthologs in *M. incognita* (Mi), *M. hapla* (Mh) and other nematodes of interest (*G. pallida,* Gp; *G. rostochiensis,* Gr; *A. suum,* As; *T. spiralis*, Ts) is indicated for each lineage. For *M. incognita*, presence of RNA-seq transcriptional support is indicated by a cross

**Fig. 7:**
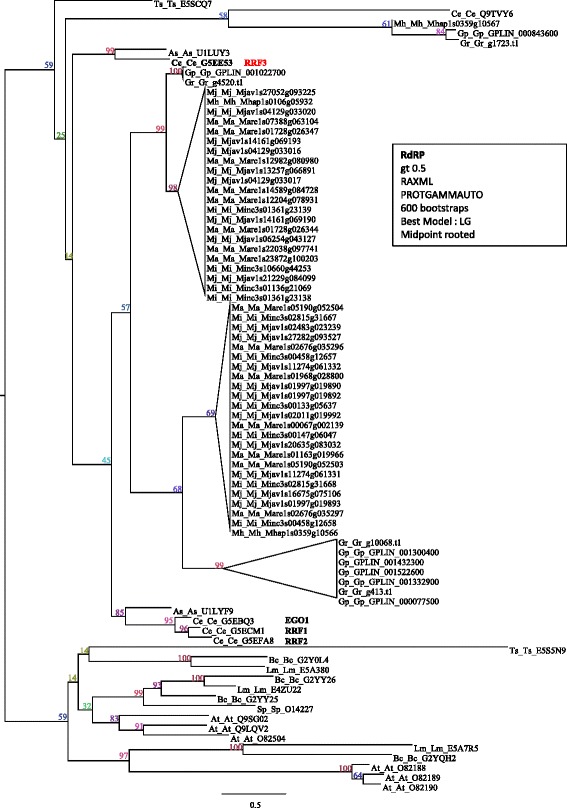
RNA-dependent RNA polymerase (RdRp) phylogenetic tree

To further investigate small ncRNA biogenesis pathways we looked for proteins from complexes that handle small ncRNA after export from the nucleus: the Drosha protein, Dicer protein, and some of the proteins binding to Drosha (PASH-1) or Dicer (DRH1/3). More especially, in *C.elegans* the ERI/DICER complex, composed of ERI-1/3/5, RRF-3, and DICER mediates RNAi processes [61]. Because siRNA can have several different origins, we also looked for more RNA-dependent RNA polymerase (RdRp) that can synthesize siRNA by copying out simple strand RNA [62]. With exception of ERI-3/5, when one gene is present in *C. elegans*, multiple co-orthologs are found in all four RKN (Table 5 and Fig. 7).

**Table 5:** Selected components of small non-coding RNA biogenesis pathways

Family	Lineage				RKN			Other nematodes		
		*C. elegans*		Mi		Mh	Gp	Gr	As	Ts
		Number	Sequences	Number	Transcriptional support	Number	Number	Number	Number	Number
ERI	ERI1	1	ERI1/O44406	3	X	1	1	1	1	0
	ERI3	1	ERI3/Q9GZI7	0		0	0	0	0	0
	ERI5	1	ERI5/Q95XS0	0	X	0	0	0	0	0
	DICER/ERI4	1	DCR1/P34529	2	X	2	2	1	0	4
RdRP	RRF3	1	RRF3/G5EE53	10	X	3	9	3	2	1
	RdRP	3	EGO1/G5EBQ3 RRF1/G5ECM1 RRF2/G5EFA8							
	DRH	1	DRH1/G5EDI8	6	X	1	0	0	0	0
		1	DRH3/Q93413	16	X	9	6	6	2	0
	Drosha	1	DRSH1/O01326	6	X	1	1	1	0	1
	DGCR8	1	PASH1/U4PRH5	3	X	1	1	1	1	1

Number of orthologs in *M. incognita* (Mi), *M. hapla* (Mh) and other nematodes of interest (*G. pallida*, Gp; *G. rostochiensis*, Gr; *A. suum*, As; *T. spiralis*, Ts) is indicated for each lineage. For *M. incognita*, presence of RNA-seq transcriptional support is indicated by a cross. ERI, Endoribonuclease; DCR, Dicer; RFF, RNA-dependent RNA polymerase Family; RdRp, RNA-dependent RNA polymerase; DRH, Dicer-related helicase; DRSH, Drosha; DGCR8, Microprocessor complex subunit DGR8/PASH, Partner or Drosha

## Discussion

Three systems including DNA methylation, post-translational histone modifications and non-coding RNA-associated gene silencing are currently considered to initiate and sustain epigenetic changes [7]. Here we used bioinformatics-driven functional annotations and literature sources to identify epigenetic machinery genes of RKN (*Meloidogyne* spp.) with a particular interest on the asexually reproducing RKN *M. incognita*.

### Absence of DNMT1 in nematodes and presence of DNMT3 restricted to *T. spiralis*

In most cases, cystosine methylation promotes heterochromatin formation and gene silencing [63]. However, cytosine methylation is not heavily present among eukaryotes. Methylcytosines are only present in cryptic proportions in *Drosophila* and totally absent in *C. elegans* [64, 65]. More generally, in nematodes, no cytosine methylation has been reported except during *T. spiralis* development [56]. When present, cytosine methylation marks are established by DNMT. In mammals, DNMT1 and DNMT3 are respectively responsible for methylation maintenance and de novo methylations [66]. DNMT2 is a tRNA methyltransferase involved in RNA-mediated epigenetic inheritance [67, 68]. However, existence of a DNMT2 DNA methyltransferase activity remains unclear and is still under debate [69]. Although potential DNMT1 were identified in nematodes [56], these proteins only share the presence of a CXXC domain (PF02008) with others DNMT1. CXXC domains are involved in non-methylCpG binding and are present in a broad-range of chromatin-binding proteins, including histone modifying enzymes (example: MLL; the Mixed Lineage Leukemia gene) or transcription factors (example: CXXC1; CXXC-type zinc finger protein 1) [70]. For this reason, CXXC domain presence alone could not be used to formally identify DNMTs. Our results suggest that DNMT1 may actually be absent in all tested nematodes, and DNMT3 restricted to *T. spiralis*, the only nematode known to possess cytosine methylation. In mammals, DNMT1 is involved in methylation maintenance and is mostly expressed during adult life. DNMT3 establishes de novo methylation and is highly expressed through development [66]. In *T. spiralis*, cytosine methylation is specifically associated with some precise developmental steps and is correlated with an increase of DNMT3 transcription. For this reason, DNMT3 presence could be sufficient to explain cytosine methylation in *T. spiralis*. Conversely, absence of DNMT3 suggests absence of cytosine methylation in other nematodes. This is consistent with the absence of methylcytosine in *C. elegans* [65] and the fact that no cytosine methylation was observed in *M. incognita* [16].

### There is no 6mA DNA demethylase counterpart in any RKN

6mA has been proposed to function as an epigenetic mark that carries heritable epigenetic information in eukaryotes, and especially in *C. elegans* [58]. Because *Meloidogyne* spp. possess no, or little, methylcytosine (5mC) DNA, we sought annotation for 6mA DNA machinery. While potential N6-adenine methyltransferases were identified in *Meloidogyne* spp., no demethylase was found. Based on the AlkB family phylogenetic tree and the identification of NMAD-1 ortholog in *G. palida*, a plant-parasitic nematode, we hypothesized that such demethylase has been lost in RKN. In absence of protein to catalyze the demethylation of methylated DNA, it is unlikely that Meloidogyne spp. display 6mA genomic DNA, unless another protein has taken over the role of 6mA demethylation.

### Histone (de)acetylation and (de)methylation machinery is conserved in RKN, and some families have expanded

The nucleosome is a structure composed by a DNA loop wrapped around two copies of each core histone (H2A, H2B, H3A, and H4), stabilized by linker histone (H1). Although core histones are extremely conserved through evolution [71], linker histone sequences differ greatly among species [72]. In *M. incognita,* 28 different core histone proteins were identified. This number of core histones is close to *C. elegans* which possesses 22 core histones. By contrast, linker histones were underepresented in *M. incognita* (two *M. incognita* H1, six *C. elegans* H1) likely because divergence in linker histone sequences makes them difficult to identify.

HAT and HDAC generally possess a wide target range and could catalyze (de)acetylation on different histone positions, or on different proteins [73]. In most cases, histone hyperacetylation results in chromatin decompaction, and thus transcription of neighboring genes. Conversely, histone hypoacetylation promotes gene silencing.

Methylation of histones is more stable than acetylation and phosphorylation marks [74]. For this reason, methylation could be considered as a “longer-term” mark which has been linked to trans-generational epigenetic heredity in *C. elegans* [75, 76]. Methylations on the positions H3K9, H3K36 and H4K20 are associated with gene inactivation while methylations on the positions H3K4 and H3K79 are associated with gene activation. These patterns of (in)activation are well conserved among evolution as they are systematically found by genome-wide approaches, in *A. thaliana* [77], *D. melanogaster* [78] and *C. elegans* [79]. All families of proteins involved in (de)acetylation and (de)methylation were found in RKN.

The fact that most *C. elegans* proteins involved in histone modifications exist in *Meloidogyne* spp. does not necessary mean that these proteins are functional. To address this question, we looked into *M. incognita* RNA-seq data for transcriptomic evidence. We found that among the 65 *C. elegans* proteins conserved in *M. incognita*, 42 possessed at least one *M. incognita* gene supported by transcriptomic data, indicating that, *C. elegans* histone (de)acetyl/(de)methylation pathways are present, conserved and probably functional in *M. incognita*.

A particular interest is transgenerational epigenetic inheritance phenomena observed in *C. elegans* and that could exist in *M. incognita*. For instance, *C. elegans* transgenerational inheritance of longevity [75] involves the H3K4-methyltransferase SET-2 which is found in four expressed genes in *M. incognita*. Another example is the transgenerational inheritance of fertility [76]. In that case, transgenerational fertility defects were caused by loss of the H3K4-demethylase SPR-5 (four genes in *M. incognita*, among which two are supported by RNA-seq) and could be accelerated by losses of some H3K4/9-methyltransferases or H3K9-demethylases including MET-2, SET-30 and JMJD-2 which were identified in *M. incognita* (respectively: 3, 2 and 3 genes). Describing histone methylation patterns in *M. incognita* (especially H3K4 and H3K9 methylation) could be of particular interest using the ChIP-seq methodology we previously developed [14].

The majority of *C. elegans* proteins could be associated by phylogeny to one *M. hapla* protein whereas a higher number of genes for each histone modifying enzyme family was observed in asexually reproducing RKN (*M. incognita, M. javanica* and *M. arenaria*).

Such gene amplifications are in accordance with the duplicated genome structure of asexually reproducing RKN in comparison to the sexual species *M. hapla* [17, 19]. Indeed, most of *C. elegans* HAT/HDAC/HMT/HDMT associate with up to three *M. incognita* genes as expected by the duplicated (likely triploid) genome structure of *M. incognita*. However, four *C. elegans* epigenetic factors (NATH-10, TAF-1, SET-16, JMJC-1) appeared particularly amplified in *M. incognita* because at least six orthologs were identified for each of these 4 genes. NATH-10 is the *C. elegans* ortholog of the human gene NAT10, a GNAT histone acetyltransferase involved in diverse processes such as telomerase regulation [80], DNA damage response [81] and cellular division [82]. In *C. elegans*, NATH-10 is a positive regulator of vulval induction. A mutation of NATH-10 increases the sperm production in hermaphrodite nematodes [83]. Six NATH-10 orthologs were found in *M. incognita* when only 1 NATH-10 ortholog was found in *M. hapla*. A biological explanation to the apparent NATH-10 expansion in *M. incognita* could be functional redundancy of feminizing genes, in the context of *M. incognita* apomictic reproduction. However, only one of the six *M. incognita* NATH-10 genes was supported by transcriptomic data, suggesting either that RNA-seq do not cover *M. incognita* NATH-10 orthologs conditions of expression, or that pseudogeneization events may have occurred. TAF-1 is the main component of the transcription factor TFIID and possesses a histone acetyltransferase activity on histones H3 and H4, and a kinase activity. While TAF-1 regulates a small amount of genes [84, 85], these genes are of crucial importance. For instance, in mammals, TAF1 regulates apoptosis and cellular cycle [85, 86]; and in *C. elegans*, TAF-1 is required for the transcription of most of the embryonic genes [87]. TAF-1 genes are amplified in *M. incognita* (13 genes). Interestingly, nine of these 13 genes were supported by transcriptomic data suggesting that most of them possess a biological role. *C. elegans* SET-16 and its human orthologs KDMT2C/D are members of the MLL-like complex, involved in H3K4 trimethylation during development and hox genes regulation [88–90]. In *C. elegans* SET-16 is also specifically involved in vulval development. *C. elegans* SET-16 is characterized by the presence of four Pfam domains: SET (PF00856), FYRC (PF05965), zf-HC5HC2H (PF13771) and FYRN (PF05964) domains. SET-16 is amplified in *M. incognita* (10 genes), including eight genes supported by transcriptomic data. However, only three *M. incognita* SET-16 orthologs possess at least one of the SET-16 Pfam domains. This observation suggests that *M. incognita* SET-16 genes are either truncated or wrongly-annotated, or that they could accomplish different functions.

In *C. elegans*, JMJC-1 is an HDMT from JMJ family involved in chromatin modifications and in stress-responses [91]. Six JMJC-1 orthologs were found in *M. incognita* and were supported by transcriptomic data. All but one *M. incognita* JMJC-1 are complete and possess the characteristic Cupin 4 domain (PF08007). The remaining one is truncated in 3′ and cannot be extended because it has reached its scaffold end. In *Meloidogyne* spp., a wide variety of stresses have been linked to male differentiation [92–96]. Interestingly, it has been proposed that sex determinism in *Meloidogyne* spp. could involve chromatin modification [97]. Because JMJC-1 is involved in both stress responses and chromatin modifications, and is amplified in asexually reproducing RKN (*M. incognita, M. javanica, M. arenaria*), it could be a candidate of choice to study in the context of sex determination. The human ortholog of JMJC-1, NO66, is known to possess a H3K4 and H3K36-specific demethylase activity [98]. For these reasons, the identification of H3K4 and/or H3K36 patterns in context of female/male development may be of interest to decipher.

### A set of HMT restricted to plant-parasitic nematodes

To refine comparative annotation of epigenetic factors in relation to the parasitic lifestyle of RKN, we included 5 other species with a parasitic lifestyle (*A. pisum, L. maculans, B. cinerea, P. falcipaum* and *S. mansoni*) trying to identify common epigenetic signatures. For instance, the same strategy succeeded to identify epigenetic factors involved in virulence of the parasite *L. maculans* based on orthology with HMT in the model fungi *Neurospora crassa* [21]*.* In *L. maculans*, effector production was shown to be regulated by the addition or deletion of chromatin marks [21].

In our study, a set of HMT was restricted to PPN (only present in *Meloidogyne* spp. and *Globodera* spp.) called thereafter PPN-SET. Because of the presence of a functional HMT SET domain and transcriptional supports, these PPN-SET may probably possess a biological role in histone methylation, in PPN. However, their roles are unknown. Because these proteins were restricted to PPN, they could be involved in plant-parasitism. Interestingly, SET proteins are known to be involved in pathogeneicity in bacteria [99]. However, our analysis should be reinforced by including additional nematode species exhibiting various lifestyles.

### Although specific Argonautes involved in endogenous siRNA and piRNA processing are absent, CSR-1 is amplified in plant-parasitic nematodes

Three classes of small ncRNA are generally distinguished in animals: miRNA, siRNA and piRNA [100]. Argonautes involved in endogenous siRNA could not be identified in PPN. Endogenous siRNA that involve ERGO-1 are maternally inherited and required for zygote development [101]. These siRNA were proposed to be involved in the control of overexpressed genes that originates from gene expansion [102]. Endogenous siRNA that involve ALG-3/4 are necessary for spermatogenesis [103]. Although in *M. incognita* males fail to reproduce with females and do not contribute to the genome of the offspring, which can explain the loss of ALG-3/4, these Argonautes are also absent in either the facultative sexual species *M. hapla*, and in the obligatory sexual species *G. pallida* and *G. rostochiensis*. For this reason, absence of endogenous siRNA processing Argonautes suggests more probably a functional diversification of siRNA pathways in PPN. In addition, PRG-1/2 could not be identified in PPN. PRG-1/2 are involved in piRNA processing and act together with CSR-1 to distinguish self (CSR-1) and non-self (PRG-1/2) during gametogenesis [104]. This observation is in accordance with previous study that found that piwi RNAs are absent outside *C. elegans* clade in nematodes, and their function may have been replaced by pathways involving nematode and non-nematode specific RdRPs [105]. Intriguingly, CSR-1 are amplified and expressed in PPN: six genes in *M. hapla*, 13 genes in *M. incognita*, seven genes in *G. pallida* and nine genes in *G. rostochiensis* were found as *C. elegans* CSR-1 orthologs. By contrast, the animal parasitic-nematode *A. suum* only possesses one CSR-1 ortholog. Amplification of genes encoding small ncRNA machinery associated proteins, such RdRPs and DRHs, is also observed in the three asexually reproducing RKN species, *M. incognita*, *M. javanica*, *M. arenaria*. Altogether, the 13 CSR-1-like, 10 RdRP and 22 DRH genes in *M. incognita* represent potential interesting epigenetic regulators.

## Conclusion

This analysis provides the first accurate, comprehensive and manually curated information about RKN proteins involved in epigenetic regulations. This analysis describes corresponding genes together with their expression levels in several developmental stages, from the asexually reproducing RKN of major agricultural importance, *M. incognita*. We believe that these functional annotations will be a valuable tool for researchers working in both the field of epigenetics, evolution, host-pathogen interaction and plant parasitism.

## Additional files

Additional file 1: **Table S1.** Reference dataset of 716 known epigenetic factors in 6 model species. (XLSX 48 kb)

Additional file 2: **Table S2.** Protein domains (Pfam) specifically associated with epigenetic factors. For each of the 6 model species, number of proteins belonging to DNMT, histones, histone modifying enzymes (Histone Acetyltransferases, HAT; Histone deacetylases, HDAC; Histone methyltransferases, HMT; Histone demethylases, HDMT; Histone kinases, HK; Histone phosphatases, HP; Histone ubiquitinyl-transferases, HUT; Histone deubiquitinases, HDU), and Argonautes are indicated, as well as the specific protein domains (Pfam) that constitute the “Pfam epigenetic factor list”. (XLSX 13 kb)

Additional file 3: **Table S3**. Epigenetic factors of *S. mansoni*. A cross indicates if the epigenetic factor has been previously identified in the literature and/or in the present study. (XLSX 15 kb)

Additional file 4: **Table S4.** Epigenetic factors of *A. pisum*. A cross indicates if the epigenetic factor has been previously identified in the literature and/or in the present study. (XLSX 25 kb)

Additional file 5: **Table S5.** Number of epigenetic factors, classified by process, identified in root-knot nematodes compared to *C. elegans*. Number of epigenetic factors for each families is detailed according to the method of identification: based on either sequence similarity (orthoMCL); or presence of specifically associated protein domain (Pfam); and after validation by phylogeny (validated). ND: Not Determined. (XLSX 15 kb)

Additional file 6: **Figure S1.** Phylogenetic tree of 6mA methyltransferases. (PPTX 81 kb)

Additional file 7: **Figure S2.** Phylogenetic tree of 6mA demethylases. (PPTX 118 kb)

Additional file 8: **Table S6.** Complete list of histones and histone modifying enzymes annotation. In the 6 model species (*C. elegans, H. sapiens, D. melanogaster, S. cerevisae, S. pombe* and *A. thaliana*), the number and the names of genes are indicated. Number of orthologs in RKN (*M. incognita, M. javanica, M. arenaria* and *M. hapla*) and other species of interest (*G. pallida, G. rostochiensis, A. suum, T. spiralis, A. pisum, A. mellifera, S. mansoni, B. cinerea, L. maculans* and *P. falciparum*) is indicated for each lineage. For *M. incogntia*, presence of RNA-seq transcriptional support is indicated by a cross. Substrates for the histone modifying enzymes of model species are indicated. Protein lineages in grey were not tested by phylogeny. ND: Not determined. (XLSX 34 kb)

Additional file 9: **Figure S3.** Phylogenetic tree of MYST family Histone Acetylransferases. (PPTX 102 kb)

Additional file 10: **Figure S4.** Phylogenetic tree of TF/NF family Histone Acetylransferases. (PPTX 83 kb)

Additional file 11: **Figure S5.** Phylogenetic tree of Histone Deacetylases. (PPTX 115 kb)

Additional file 12: **Figure S6.** Phylogenetic tree of PRMT family Histone Methyltransferases. (PPTX 96 kb)

Additional file 13: **Figure S7.** Phylogenetic tree of Euchromatin SET Histone Methyltransferases 1. (PPTX 66 kb)

Additional file 14: **Figure S8.** Phylogenetic tree of Euchromatin SET Histone Methyltransferases 2. (PPTX 111 kb)

Additional file 15: **Figure S9.** Phylogenetic tree of Heterochromatin SET Histone Methyltransferases. (PPTX 103 kb)

Additional file 16: **Figure S10.** Phylogenetic tree of Histone Demethylases. (PPTX 119 kb)

Additional file 17: **Figure S11.** Phylogenetic tree of WAGO Argonautes. Putative Argonaute proteins from 7 nematodes (*C. elegans, M. incognita, M. hapla, G. pallida, G. rostochiensis, A. suum* and *T. spiralis*) were selected to build the whole argonaute tree (Fig. 3). From this whole Argonaute tree, three branches corresponded to WAGO (cytoplasmic WAGO, nuclear WAGO, self-recongnition pathway WAGO) Argonautes. *M. incognita* and *M. hapla* are colored in green. *C. elegans* proteins are colored in red. (PPTX 165 kb)

Additional file 18: **Figure S12.** Phylogenetic tree of PIWI Argonautes. (PPTX 83 kb)

## Abbreviations

AlkB: Alkylated DNA repair protein B
CARM1: Histone-arginine methyltransferase 1
CBP/P300: CREB-binding protein and P300
CMT: Cromomethylase
DAMT: DNA N6-methyltransferase
DNMT: DNA-methyltransferase
DOT1: Disruptor of telomeric 1
DRH: Dicer related helicase
GNAT: Gcn5-related N-acetyltransferase
HAT: Histone acetyltransferase
HDAC: Histone deacetylase
HDMT: Histone demethylase
HK: Histone kinase
HMT: Histone methyltransferase
HP: Histone phosphatase
HUT: Histone ubiquitin-transferase
JMJ: Jumonji demethylase
LSD: Lysine-specific demethylase
miRNA: Micro RNA
MYST: MOZ/Ybf2/Sas3/Sas2/TIP60
ncRNA: non coding RNA
NMAD: DNA N6-methyl adenine demethylase
PCAF: P300/CBP-associated factor
piRNA: PIWI-interacting RNA
PPN: Plant-parasitic nematodes
PRMT: Protein arginin methyltransferase
PTMs: Post-translational modifications
RdRp: RNA-dependent RNA polymerase
RKN: Root-knot nematodes
RPKM: Reads per kilobase of transcript per million mapped reads
SET: Suppressor of variegation/enhancer of zeste/trithorax
siRNA: Small interfering RNA
WAGO: Worm-specific argonaute
