# Supplementary material for: Genome-wide expert annotation of the epigenetic machinery of the plant-parasitic nematodes Meloidogyne spp., with a focus on the asexually reproducing species
Source: BMC Genomics. 2018 May 3;19:321. doi: 10.1186/s12864-018-4686-x (PMC5934874; doi:10.1186/s12864-018-4686-x)
Supplement: Supplementary file 6 — Figure S1. Phylogenetic tree of 6mA methyltransferases. (PPTX 81 kb) [file 12864_2018_4686_MOESM6_ESM.pptx]

## Slide 1
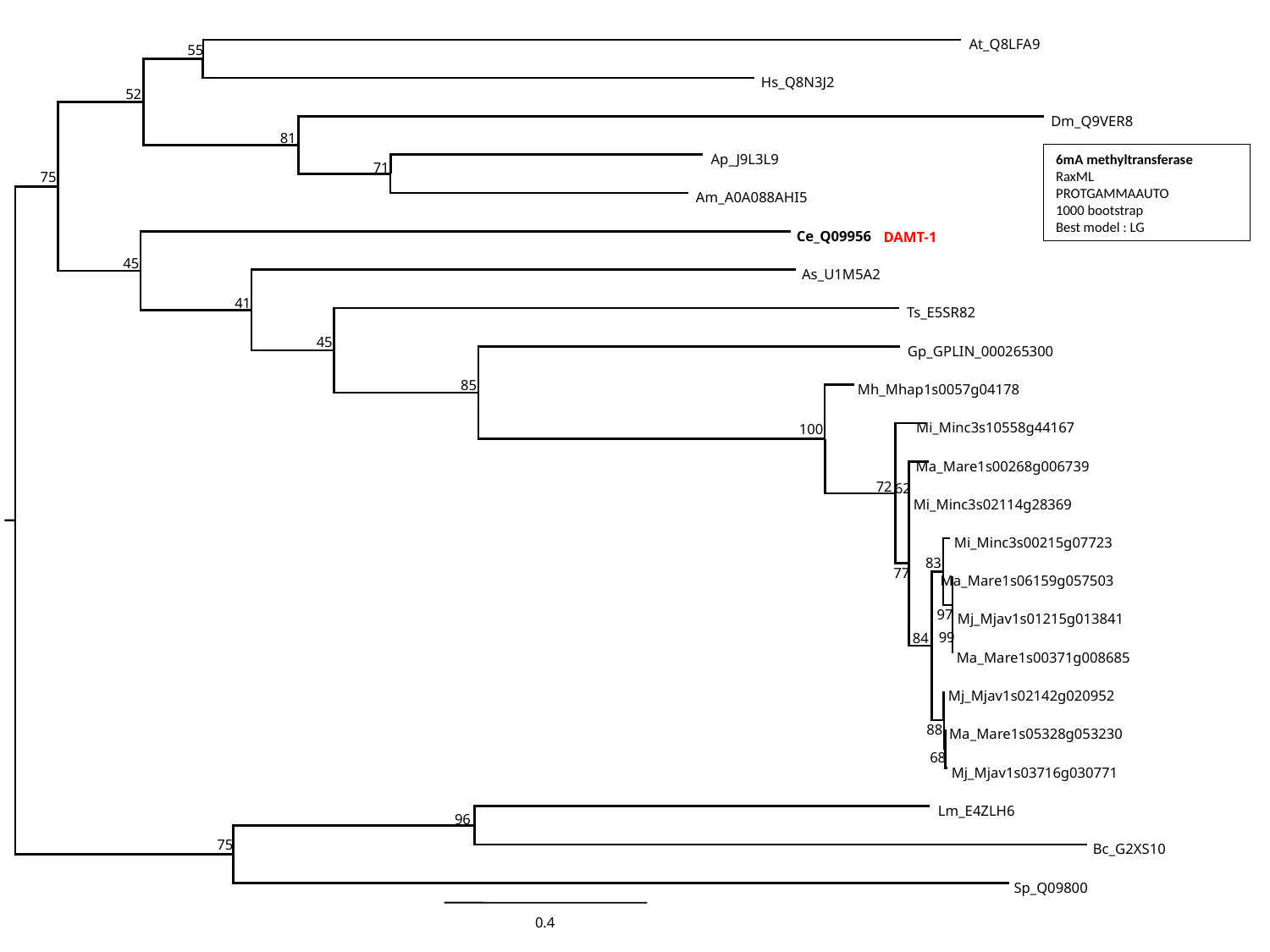

At_Q8LFA9
55
Hs_Q8N3J2
52
Dm_Q9VER8
81
6mA methyltransferase
RaxML
PROTGAMMAAUTO
1000 bootstrap
Best model : LG
Ap_J9L3L9
71
75
Am_A0A088AHI5
Ce_Q09956
DAMT-1
45
As_U1M5A2
41
Ts_E5SR82
45
Gp_GPLIN_000265300
85
Mh_Mhap1s0057g04178
Mi_Minc3s10558g44167
100
Ma_Mare1s00268g006739
72
62
Mi_Minc3s02114g28369
Mi_Minc3s00215g07723
83
77
Ma_Mare1s06159g057503
97
Mj_Mjav1s01215g013841
99
84
Ma_Mare1s00371g008685
Mj_Mjav1s02142g020952
88
Ma_Mare1s05328g053230
68
Mj_Mjav1s03716g030771
Lm_E4ZLH6
96
75
Bc_G2XS10
Sp_Q09800
0.4
