# Supplementary material for: Genome-wide expert annotation of the epigenetic machinery of the plant-parasitic nematodes Meloidogyne spp., with a focus on the asexually reproducing species
Source: BMC Genomics. 2018 May 3;19:321. doi: 10.1186/s12864-018-4686-x (PMC5934874; doi:10.1186/s12864-018-4686-x)
Supplement: Supplementary file 7 — Figure S2. Phylogenetic tree of 6mA demethylases. (PPTX 118 kb) [file 12864_2018_4686_MOESM7_ESM.pptx]

## Slide 1
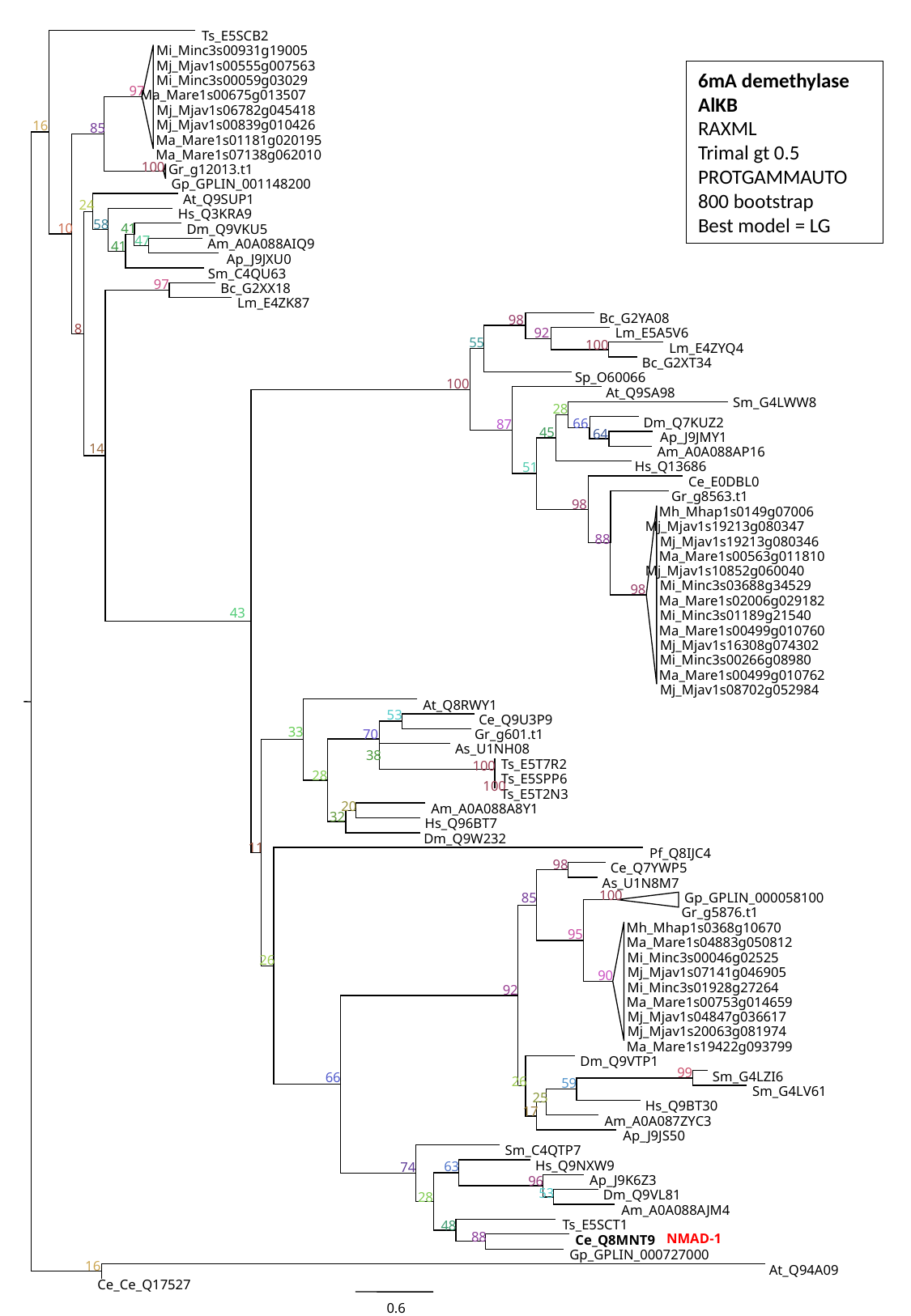

Ts_E5SCB2
Mj_Mjav1s00555g007563
Mi_Minc3s00059g03029
Mj_Mjav1s00839g010426
Ma_Mare1s01181g020195
Gr_g12013.t1
Gp_GPLIN_001148200
Bc_G2XX18
Lm_E4ZK87
Bc_G2YA08
At_Q9SA98
Ma_Mare1s00563g011810
Mj_Mjav1s10852g060040
Mi_Minc3s03688g34529
Mi_Minc3s01189g21540
Ce_Q9U3P9
Ts_E5T2N3
Pf_Q8IJC4
Ce_Q7YWP5
Gp_GPLIN_000058100
Mh_Mhap1s0368g10670
Mi_Minc3s00046g02525
Mj_Mjav1s07141g046905
Mj_Mjav1s20063g081974
Sm_G4LV61
Hs_Q9NXW9
Dm_Q9VL81
Ts_E5SCT1
0.6
Mi_Minc3s00931g19005
6mA demethylase
AlKB
RAXML
Trimal gt 0.5
PROTGAMMAUTO
800 bootstrap
Best model = LG
97
Ma_Mare1s00675g013507
Mj_Mjav1s06782g045418
16
85
Ma_Mare1s07138g062010
100
At_Q9SUP1
24
Hs_Q3KRA9
58
10
41
Dm_Q9VKU5
47
Am_A0A088AIQ9
41
Ap_J9JXU0
Sm_C4QU63
97
98
8
92
Lm_E5A5V6
55
100
Lm_E4ZYQ4
Bc_G2XT34
Sp_O60066
100
Sm_G4LWW8
28
Dm_Q7KUZ2
66
87
45
64
Ap_J9JMY1
14
Am_A0A088AP16
Hs_Q13686
51
Ce_E0DBL0
Gr_g8563.t1
98
Mh_Mhap1s0149g07006
Mj_Mjav1s19213g080347
88
Mj_Mjav1s19213g080346
98
Ma_Mare1s02006g029182
43
Ma_Mare1s00499g010760
Mj_Mjav1s16308g074302
Mi_Minc3s00266g08980
Ma_Mare1s00499g010762
Mj_Mjav1s08702g052984
At_Q8RWY1
53
33
70
Gr_g601.t1
As_U1NH08
38
Ts_E5T7R2
100
28
Ts_E5SPP6
100
20
Am_A0A088A8Y1
32
Hs_Q96BT7
Dm_Q9W232
11
98
As_U1N8M7
100
85
Gr_g5876.t1
95
Ma_Mare1s04883g050812
26
90
Mi_Minc3s01928g27264
92
Ma_Mare1s00753g014659
Mj_Mjav1s04847g036617
Ma_Mare1s19422g093799
Dm_Q9VTP1
99
Sm_G4LZI6
66
26
59
25
Hs_Q9BT30
17
Am_A0A087ZYC3
Ap_J9JS50
Sm_C4QTP7
63
74
Ap_J9K6Z3
96
53
28
Am_A0A088AJM4
48
88
NMAD-1
Ce_Q8MNT9
Gp_GPLIN_000727000
16
At_Q94A09
Ce_Ce_Q17527
