# Supplementary figures and images for: Genome-wide expert annotation of the epigenetic machinery of the plant-parasitic nematodes Meloidogyne spp., with a focus on the asexually reproducing species
Source: BMC Genomics. 2018 May 3;19:321. doi: 10.1186/s12864-018-4686-x (PMC5934874; doi:10.1186/s12864-018-4686-x)

## Slide 1
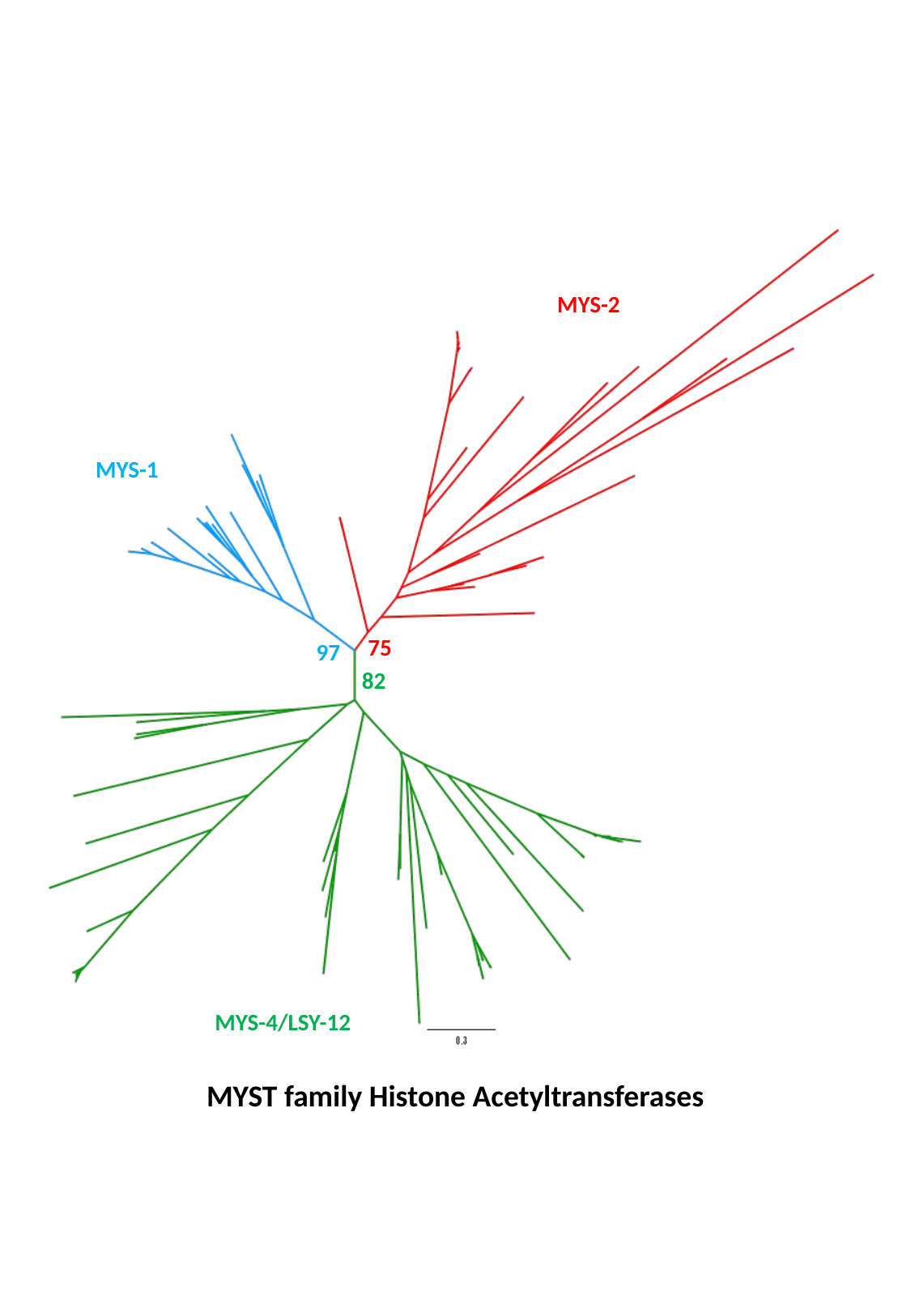

MYS-2
MYS-1
75
97
82
MYS-4/LSY-12
MYST family Histone Acetyltransferases

Supplement: Supplementary file 9 — Figure S3. Phylogenetic tree of MYST family Histone Acetylransferases. (PPTX 102 kb) [file 12864_2018_4686_MOESM9_ESM.pptx]

## Slide 1
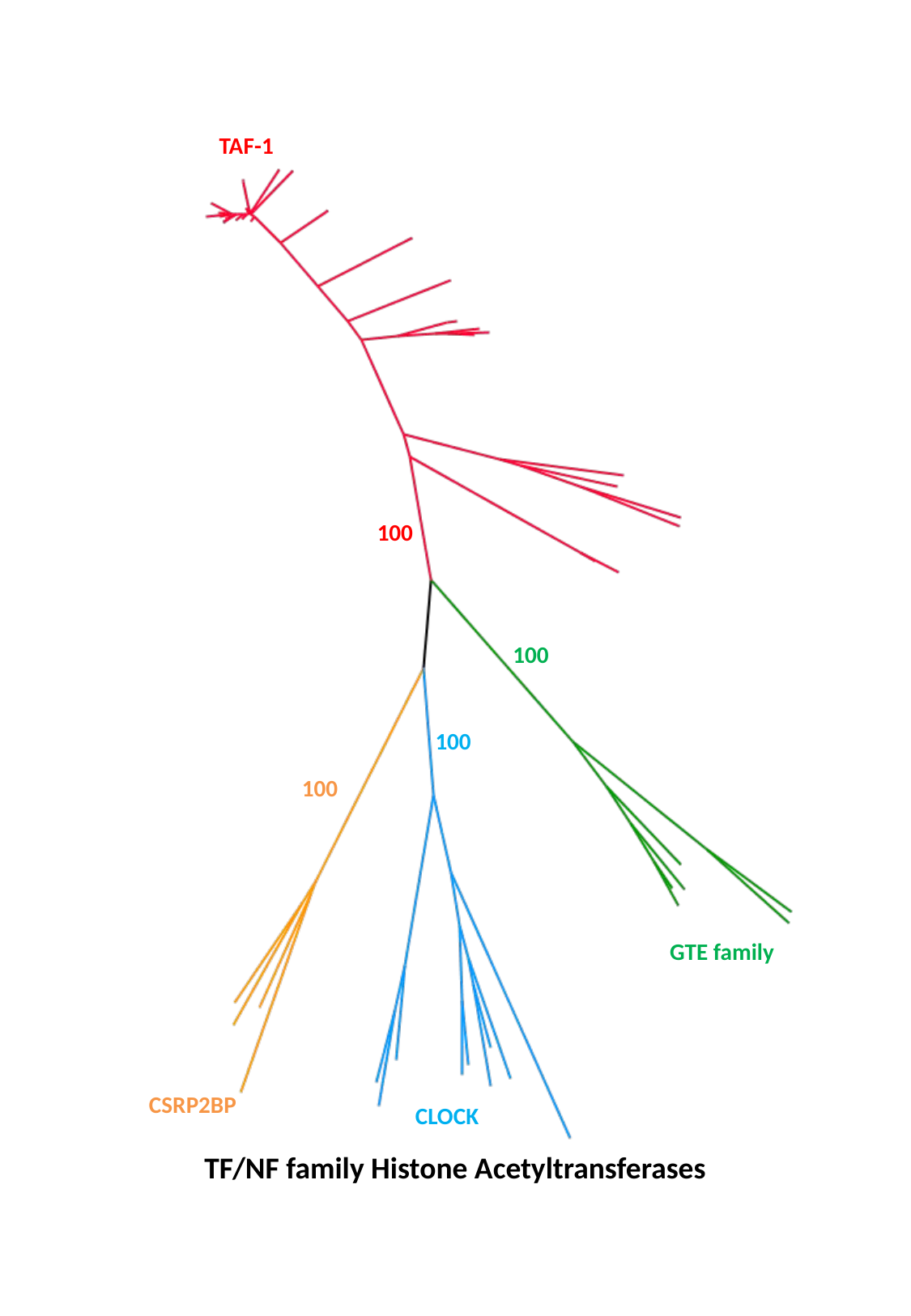

TAF-1
100
100
100
100
GTE family
CSRP2BP
CLOCK
TF/NF family Histone Acetyltransferases

Supplement: Supplementary file 10 — Figure S4. Phylogenetic tree of TF/NF family Histone Acetylransferases. (PPTX 83 kb) [file 12864_2018_4686_MOESM10_ESM.pptx]

## Slide 1
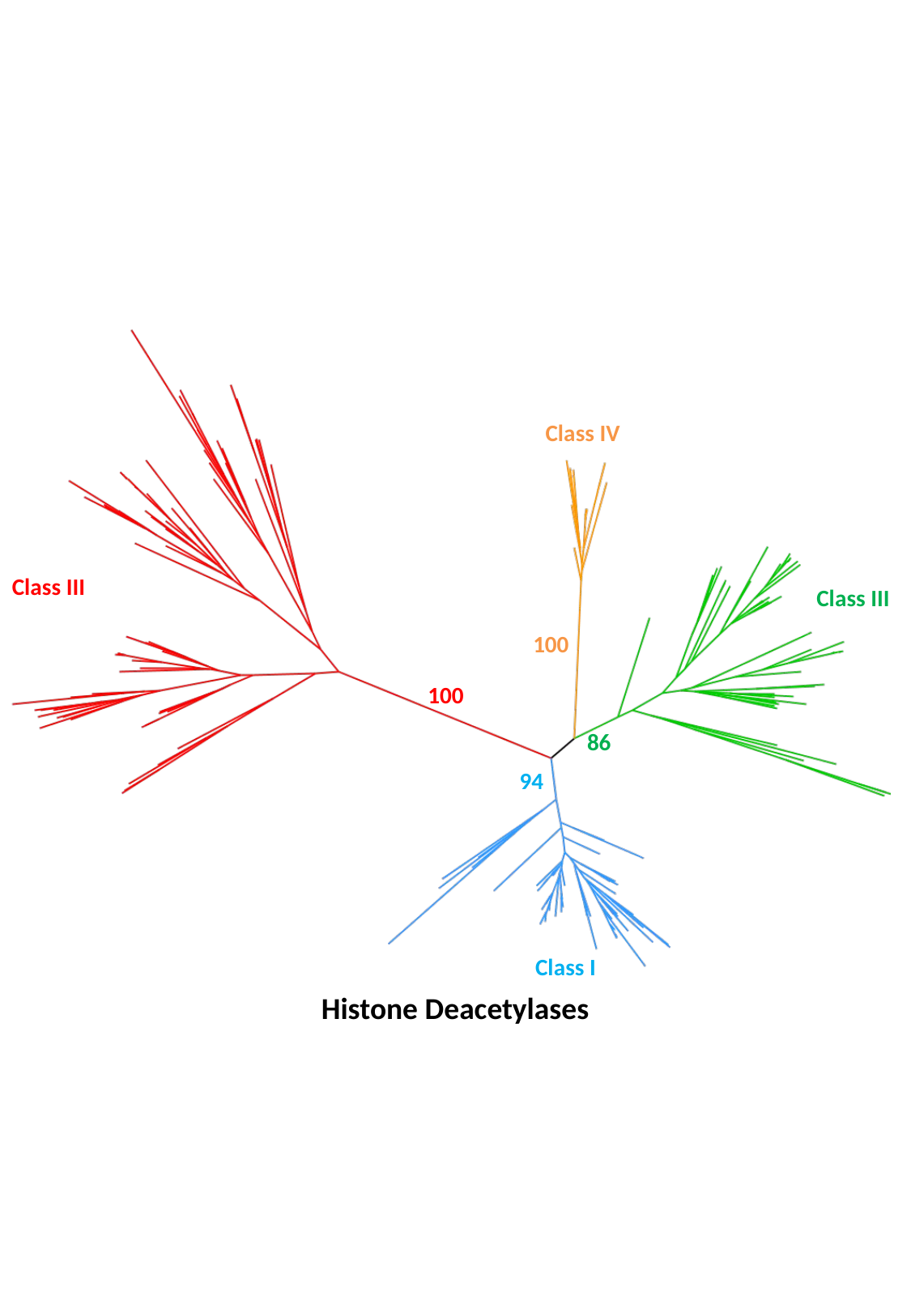

Class IV
Class III
Class III
100
100
86
94
Class I
Histone Deacetylases

Supplement: Supplementary file 11 — Figure S5. Phylogenetic tree of Histone Deacetylases. (PPTX 115 kb) [file 12864_2018_4686_MOESM11_ESM.pptx]

## Slide 1
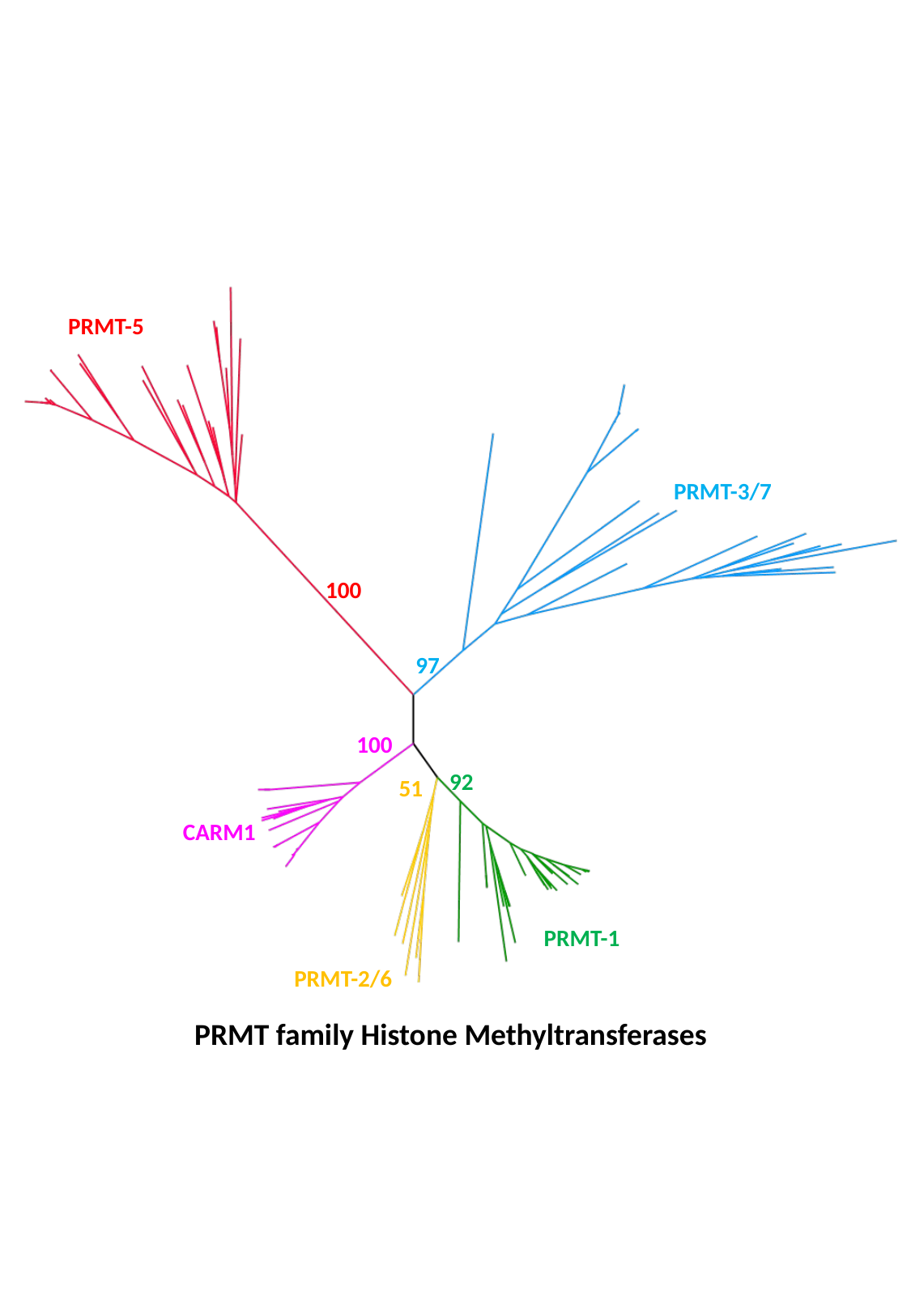

PRMT-5
PRMT-3/7
100
97
100
92
51
CARM1
PRMT-1
PRMT-2/6
PRMT family Histone Methyltransferases

Supplement: Supplementary file 12 — Figure S6. Phylogenetic tree of PRMT family Histone Methyltransferases. (PPTX 96 kb) [file 12864_2018_4686_MOESM12_ESM.pptx]

## Slide 1
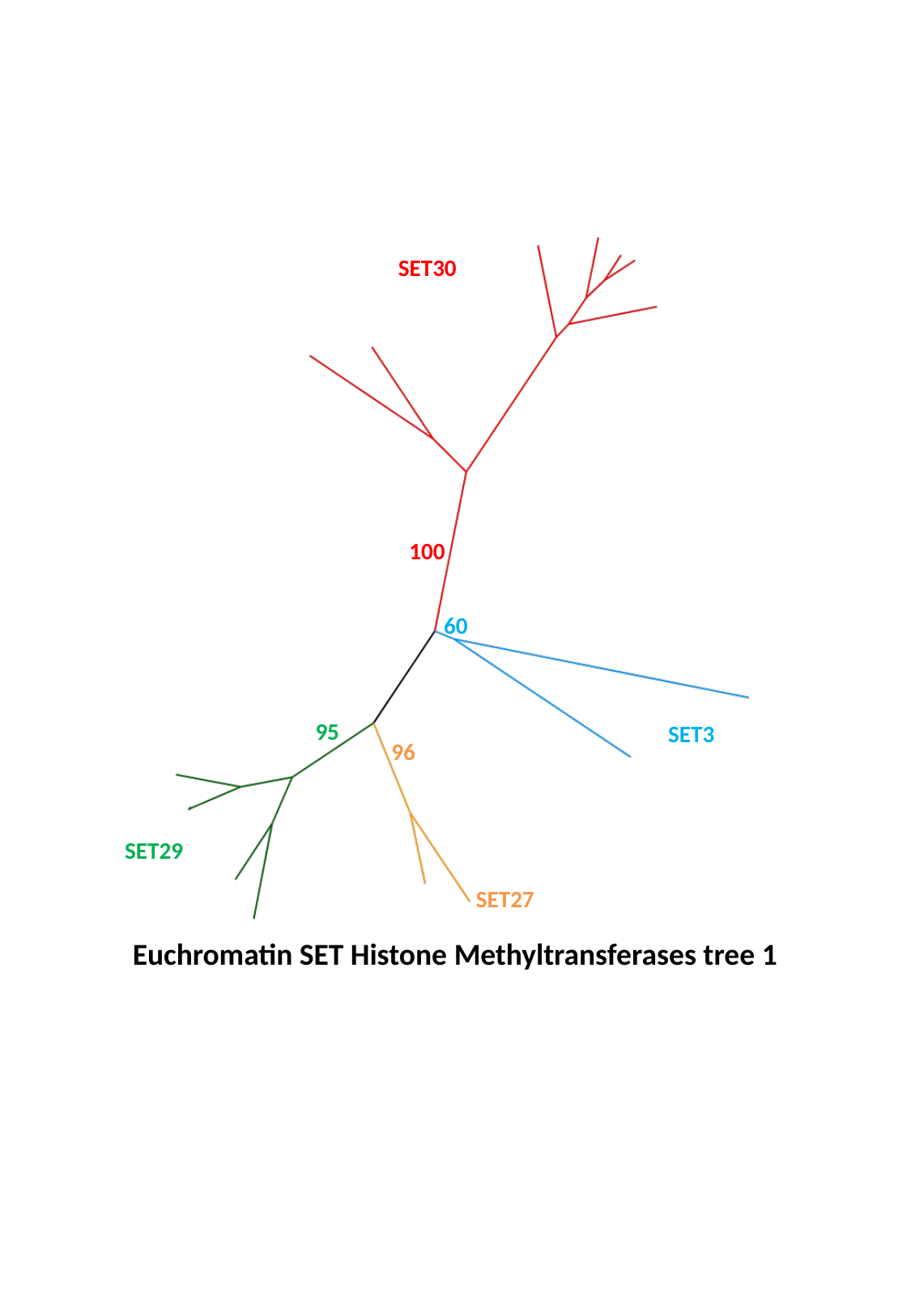

SET30
100
60
95
SET3
96
SET29
SET27
Euchromatin SET Histone Methyltransferases tree 1

Supplement: Supplementary file 13 — Figure S7. Phylogenetic tree of Euchromatin SET Histone Methyltransferases 1. (PPTX 66 kb) [file 12864_2018_4686_MOESM13_ESM.pptx]

## Slide 1
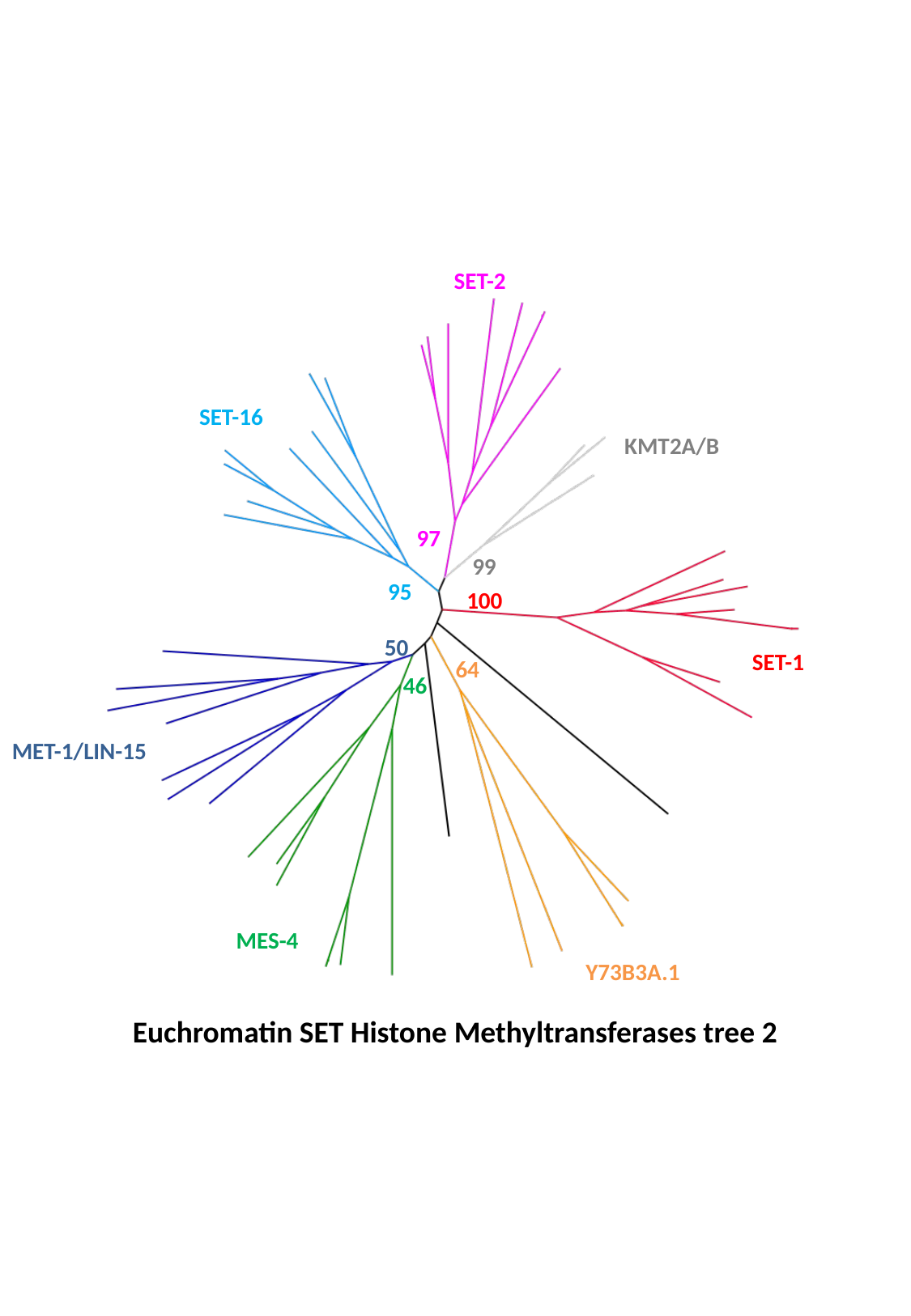

SET-2
SET-16
KMT2A/B
97
99
95
100
50
SET-1
64
46
MET-1/LIN-15
MES-4
Y73B3A.1
Euchromatin SET Histone Methyltransferases tree 2

Supplement: Supplementary file 14 — Figure S8. Phylogenetic tree of Euchromatin SET Histone Methyltransferases 2. (PPTX 111 kb) [file 12864_2018_4686_MOESM14_ESM.pptx]

## Slide 1
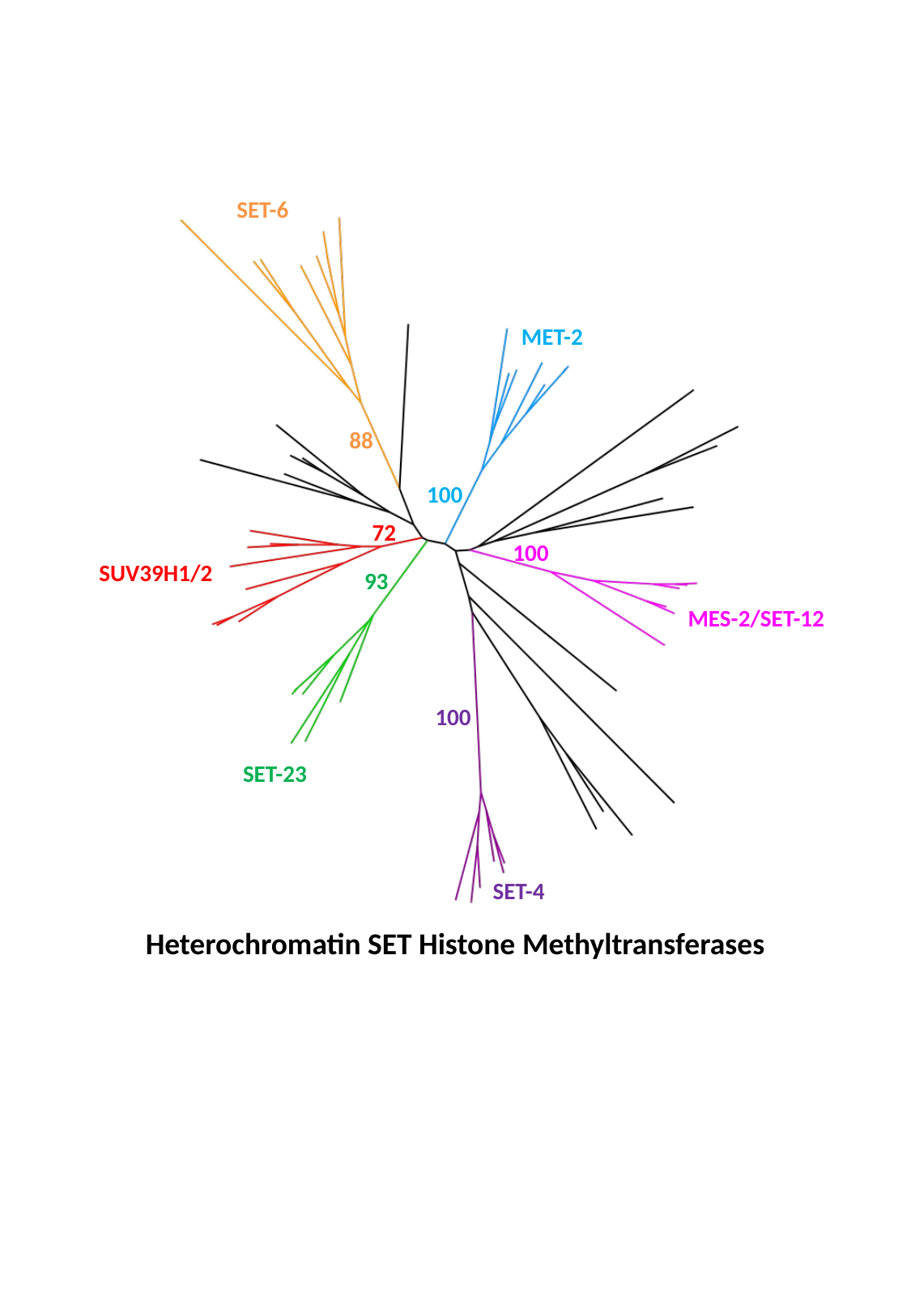

SET-6
MET-2
88
100
72
100
SUV39H1/2
93
MES-2/SET-12
100
SET-23
SET-4
Heterochromatin SET Histone Methyltransferases

Supplement: Supplementary file 15 — Figure S9. Phylogenetic tree of Heterochromatin SET Histone Methyltransferases. (PPTX 103 kb) [file 12864_2018_4686_MOESM15_ESM.pptx]

## Slide 1
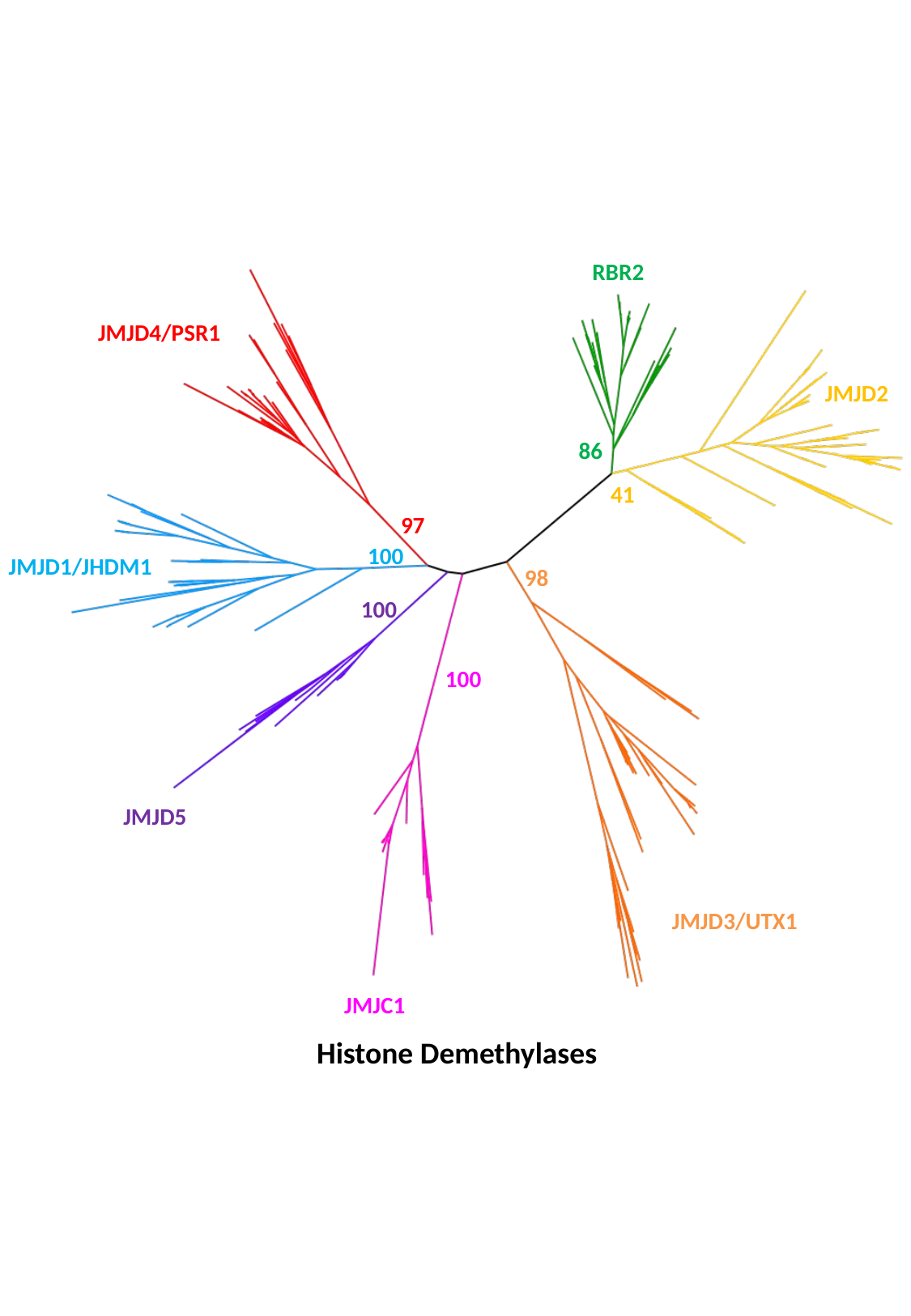

RBR2
JMJD4/PSR1
JMJD2
86
41
97
100
JMJD1/JHDM1
98
100
100
JMJD5
JMJD3/UTX1
JMJC1
Histone Demethylases

Supplement: Supplementary file 16 — Figure S10. Phylogenetic tree of Histone Demethylases. (PPTX 119 kb) [file 12864_2018_4686_MOESM16_ESM.pptx]

## Slide 1
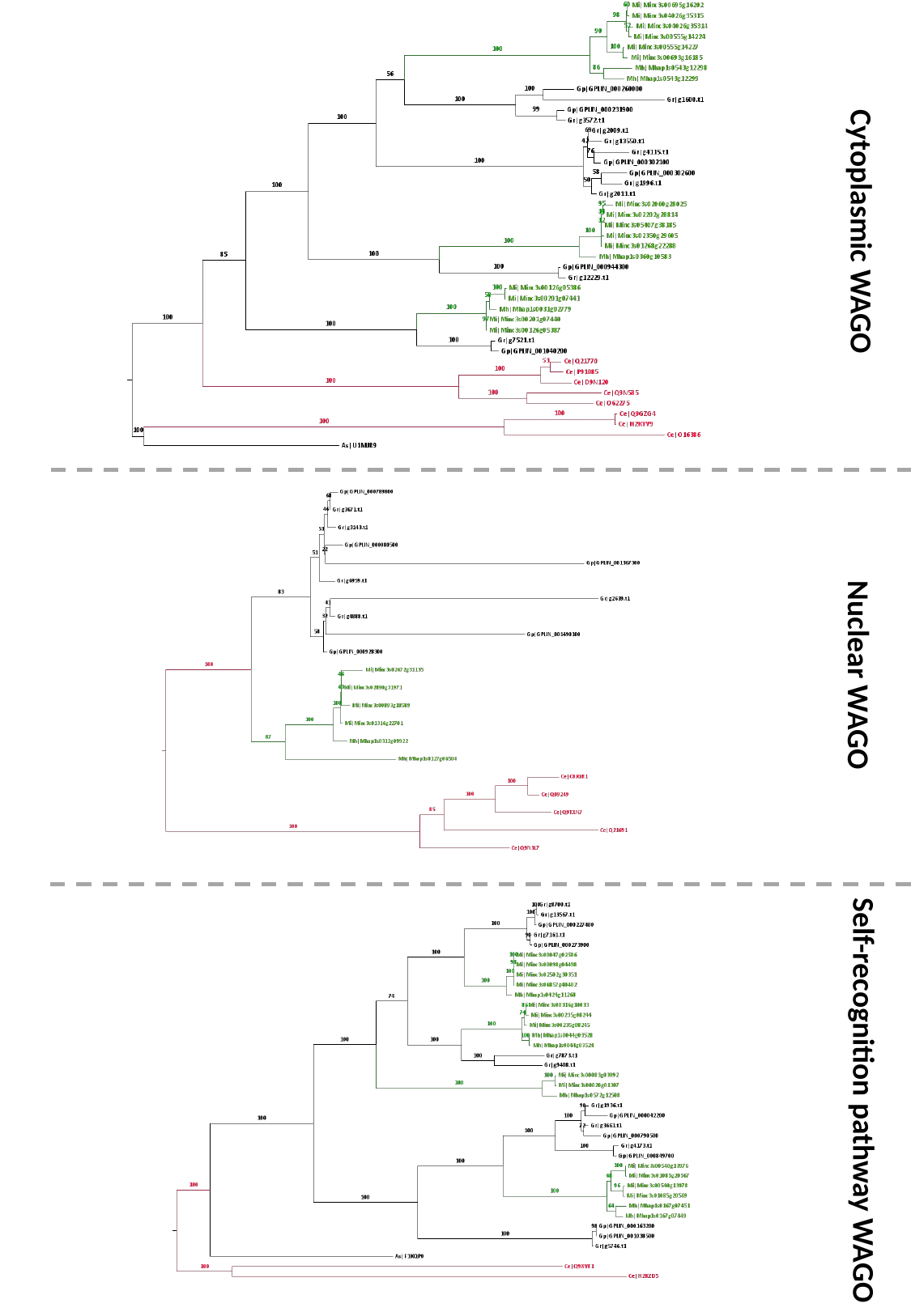

Cytoplasmic WAGO
Nuclear WAGO
Self-recognition pathway WAGO

Supplement: Supplementary file 17 — Figure S11. Phylogenetic tree of WAGO Argonautes. Putative Argonaute proteins from 7 nematodes (C. elegans, M. incognita, M. hapla, G. pallida, G. rostochiensis, A. suum and T. spiralis) were selected to build the whole argonaute tree (Fig. 3). From this whole Argonaute tree, three branches corresponded to WAGO (cytoplasmic WAGO, nuclear WAGO, self-recongnition pathway WAGO) Argonautes. M. incognita and M. hapla are colored in green. C. elegans proteins are colored in red. (PPTX 165 kb) [file 12864_2018_4686_MOESM17_ESM.pptx]
