# Supplementary material for: Genome-wide expert annotation of the epigenetic machinery of the plant-parasitic nematodes Meloidogyne spp., with a focus on the asexually reproducing species
Source: BMC Genomics. 2018 May 3;19:321. doi: 10.1186/s12864-018-4686-x (PMC5934874; doi:10.1186/s12864-018-4686-x)
Supplement: Supplementary file 18 — Figure S12. Phylogenetic tree of PIWI Argonautes. (PPTX 83 kb) [file 12864_2018_4686_MOESM18_ESM.pptx]

## Slide 1
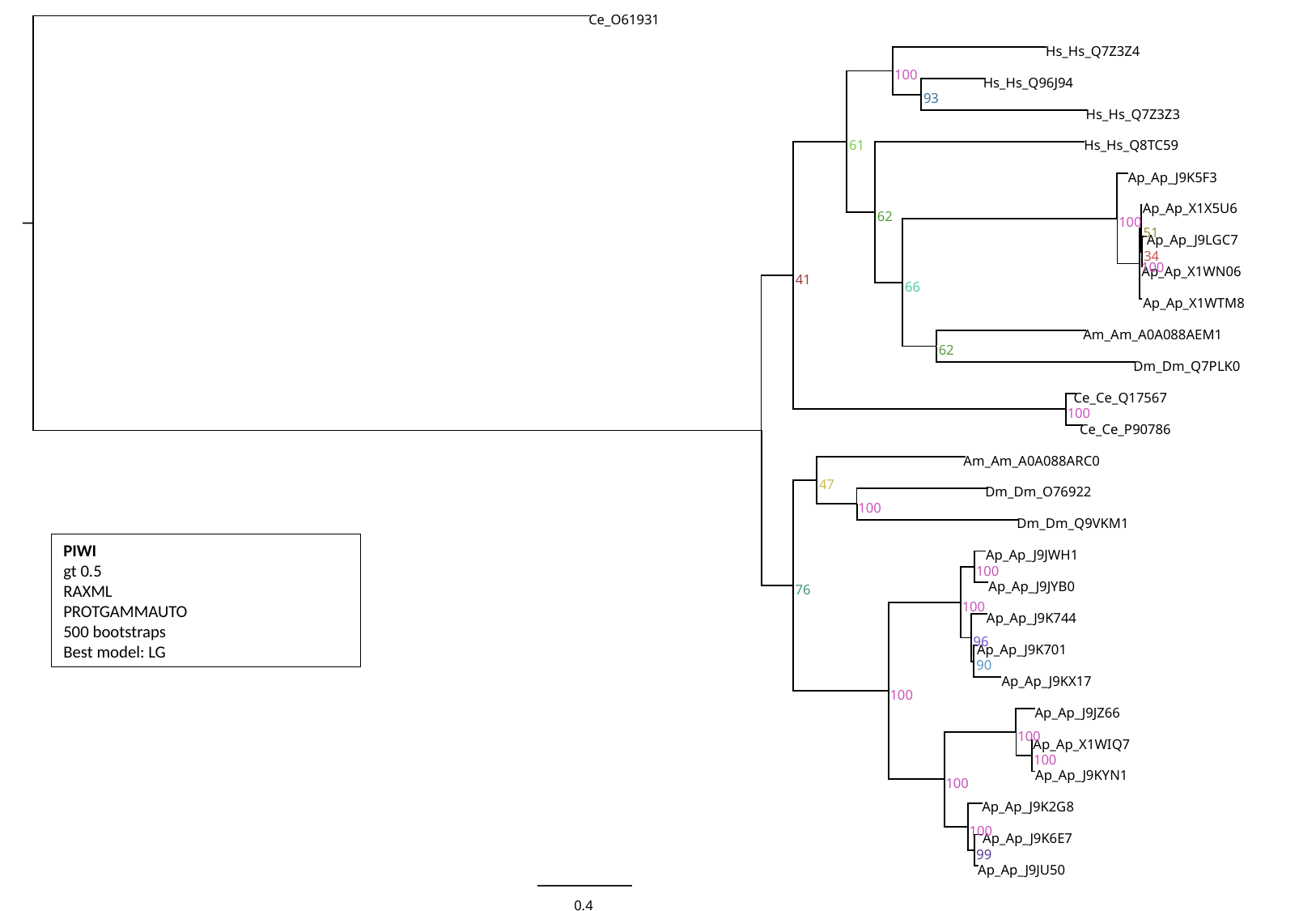

Ce_O61931
Hs_Hs_Q7Z3Z4
100
Hs_Hs_Q96J94
93
Hs_Hs_Q7Z3Z3
61
Hs_Hs_Q8TC59
Ap_Ap_J9K5F3
Ap_Ap_X1X5U6
62
100
51
Ap_Ap_J9LGC7
34
100
Ap_Ap_X1WN06
41
66
Ap_Ap_X1WTM8
Am_Am_A0A088AEM1
62
Dm_Dm_Q7PLK0
Ce_Ce_Q17567
100
Ce_Ce_P90786
Am_Am_A0A088ARC0
47
Dm_Dm_O76922
100
Dm_Dm_Q9VKM1
PIWI
gt 0.5
RAXML
PROTGAMMAUTO
500 bootstraps
Best model: LG
Ap_Ap_J9JWH1
100
Ap_Ap_J9JYB0
76
100
Ap_Ap_J9K744
96
Ap_Ap_J9K701
90
Ap_Ap_J9KX17
100
Ap_Ap_J9JZ66
100
Ap_Ap_X1WIQ7
100
Ap_Ap_J9KYN1
100
Ap_Ap_J9K2G8
100
Ap_Ap_J9K6E7
99
Ap_Ap_J9JU50
0.4
